# mRNA processing in cancer immunotherapy: emerging targets, resistance mechanisms, and therapeutic opportunities

**DOI:** 10.3389/fimmu.2026.1889279

**Published:** 2026-09-16

**Authors:** Yunus Yukselten, Haseeb Ahmad, Mohammed Shoultout, Uzair Iqbal, Faizan Masood, Richard E. Sutton

**Affiliations:** Section of Infectious Diseases, Department of Internal Medicine, Yale University, New Haven, CT, United States

**Keywords:** 3′UTR remodeling, cancer immunotherapy, chemokines, epitranscriptomics, immunopeptidomics, mRNA processing, RNA editing, splicing-derived neoantigens

## Abstract

Cancer immunotherapy has improved outcomes across many tumor types, but primary and acquired resistance, tumor heterogeneity and a shortage of safe targets remain unresolved. Part of this gap arises because tumor cells evade immune recognition not only through genomic mutation but also through post-transcriptional mRNA processing, a network comprising 5′ capping, splicing, alternative polyadenylation (APA), RNA editing, epitranscriptomic modification, nonsense-mediated decay (NMD), RNA stability and translational control. These processes govern antigen presentation, transcript degradation, checkpoint expression and the suppression of innate immune sensing. Here we examine how mRNA processing can extend the target space of cancer immunotherapy. We consider alternative splicing as a source of tumor-specific isoforms, public neoantigens and chimeric antigen receptor (CAR) or T-cell receptor (TCR)-based targets; the effect of APA and 3′UTR remodeling on checkpoints such as PD-L1; the role of m6A, ac4C and other epitranscriptomic marks in antigen presentation, interferon signaling and the tumor microenvironment; the contribution of ADAR1-mediated editing to immunotherapy resistance through suppressed dsRNA and Z-RNA sensing; and the function of NMD as an antigen filter. We also review the discovery technologies that make these targets accessible, and assess the current state of clinical translation, including agent development, target specificity, patient selection, biomarkers and safety.

mRNA processing-derived targets offer new sources of antigens, biomarkers and combination strategies in tumors with low mutational burden or refractory to checkpoint blockade. Realizing this will require validation of RNA-level candidates at the protein and HLA-peptide level, together with attention to tumor-normal specificity, HLA restriction, tumor heterogeneity and toxicity.

## 1 Introduction

Immunotherapy has transformed cancer treatment by demonstrating that the crosstalk between tumor cells and the immune system can be therapeutically targeted. Immune checkpoint inhibitors, adoptive cell therapies, cancer vaccines, bispecific antibodies, and mRNA-based platforms can generate durable responses across tumor types. But these effects are not all-encompassing. Many tumors are initially unresponsive or acquire resistance after an initial response, often through antigen loss, defective HLA/MHC presentation, an immunosuppressive tumor microenvironment, T-cell dysfunction, and altered checkpoint regulation (1, 2).

How the immune system recognizes a tumor cell is closely linked to how genomic information is processed at the RNA and protein levels. A conceptual overview of how mRNA processing links tumor genomics to immune visibility, immune escape, and therapeutic opportunity is shown in **Figure 1**. Pre-mRNA maturation, alternative splicing, alternative polyadenylation, RNA editing, RNA modifications, nonsense-mediated decay or NMD, and mRNA stability can generate distinct isoforms, transcripts with different 3′UTR structures, altered protein products with new antigenic peptides from the same genome (3–6). mRNA processing can therefore be viewed as a dynamic intermediate layer between genetic alterations and immunological outcome.

**Figure 1:**
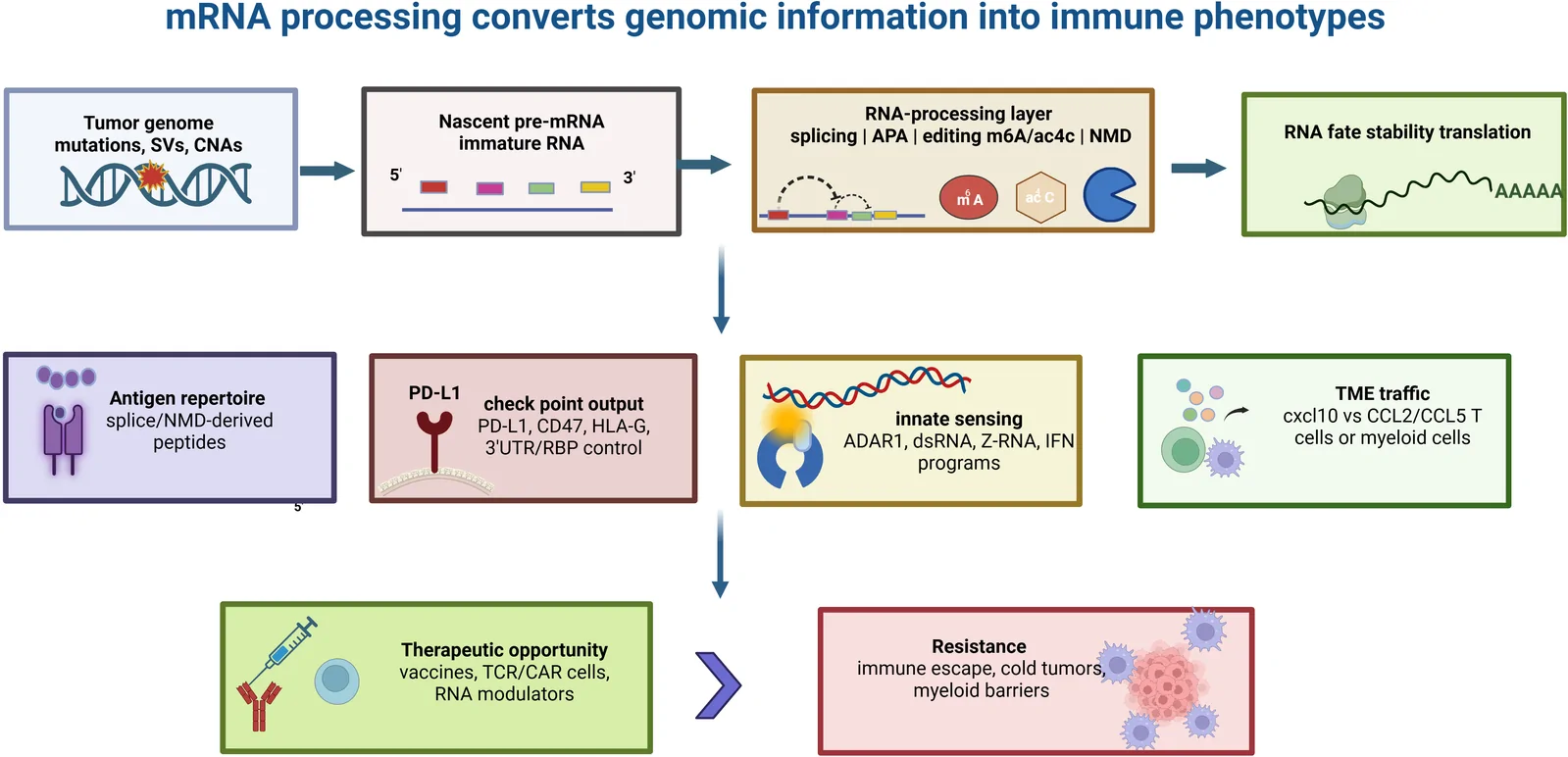
Schematic summarizes how tumor genomic information is translated into immune-relevant phenotypes by post-transcriptional RNA processing. Genomic alterations (mutations, structural variants and copy number alterations) are transcribed into nascent pre-mRNA and are then sculpted by RNA processing mechanisms, including alternative splicing, alternative polyadenylation, RNA editing, m6A/ac4C modification and nonsense-mediated decay. These processes affect RNA fate including transcript stability and translation and produce downstream immune outputs including splice- or NMD-derived antigen repertoires, checkpoint regulation, innate RNA sensing programs, and tumor microenvironment trafficking. Depending on cellular and microenvironmental context, these RNA level outputs can translate into therapeutic opportunities such as vaccines, TCR/CAR-based therapies, and RNA modulators or resistance biology via immune escape, cold tumors, and myeloid barriers.

This post-transcriptional layer has become increasingly relevant to immunotherapy. Broad, shared neoantigens generated by aberrant alternative splicing have been identified across cancer types, supporting splicing-derived targets as a source of antigens for T-cell-based therapies (7, 8). NMD inhibition has also been reported to stabilize aberrant transcripts, uncover non-canonical neoantigens and improve checkpoint blockade efficacy (9, 10). At the same time, mRNA processing contributes to immune escape. Structural variation in the PD-L1 3′UTR can stabilize PD-L1 transcripts and support immune evasion (11). Loss of ADAR1 can enhance endogenous dsRNA sensing, sensitize tumors to immune checkpoint blockade and, in some contexts, overcome PD-1 resistance (12–18). These findings suggest that tumor cells escape immune surveillance not only by suppressing T cells, but also by silencing antiviral-like RNA alarms inside the cell.

Here we address the role of mRNA processing in cancer immunotherapy target discovery. We discuss alternative splicing, alternative polyadenylation, epitranscriptomic modification, RNA editing, and NMD in relation to tumor immunogenicity and immune escape. We also review bulk and single-cell RNA-seq, long-read sequencing, spatial transcriptomics, immunopeptidomics and functional validation strategies used to identify targets derived from these mechanisms.

Target discovery in cancer immunotherapy requires going beyond DNA mutation burden, tumor antigen expression and checkpoint protein levels. RNA fate after transcription is directly associated with how the tumor is perceived by the immune system. This post-transcriptional network may be classified into mechanisms that generate new antigenic products and isoforms, methods that rebalance checkpoint and immune-regulatory protein expression, processes that affect innate immune sensing and translational control, and circumstances that translate molecular changes into immune-cell composition through chemokine programs and the tumor microenvironment (11, 12, 19–21). Thus, mRNA processing-derived targets can serve as antigens, biomarkers, and therapeutic intervention points, especially for tumors where classical mutation-derived neoantigen discovery is insufficient. For clinical use, however, an RNA-level target must be reflected at the protein or peptide level, presented by HLA/MHC, safe in normal tissues, and able to generate a functional T-cell response (17, 18, 22).

### 1.1 Alternative splicing and immunogenic tumor isoforms

Alternative splicing is one of the strongest post-transcriptional mechanisms through which cancer cells generate different transcripts and protein isoforms from the same encoded information in the genome. Through alternative exon usage, exon skipping, alternative splice-site selection, intron retention and cryptic splice junction formation, tumor cells can produce RNA and protein isoforms that are absent or expressed at very low levels in normal tissues (23).

For immunotherapy, the main aim is not simply that alternative splicing increases protein diversity. It can also produce tumor-specific peptide sequences, novel exon-exon junctions, aberrant translation products from retained introns, or isoform-specific targets enriched at the cell surface or in the extracellular matrix. These targets can be grouped into HLA/MHC-presented peptides and surface or matrix isoforms suitable for chimeric antigen receptor or CAR T cell or antibody-based targeting (7, 13). Pan-cancer analyses have revealed increased alternative splicing and many neojunctions in tumor samples compared with normal tissue (24). Immunopeptidomics has shown that retained intron-derived neoepitopes can be presented on MHC-I, supporting the idea that RNA-level splicing errors can result in real antigen-presentation events (25).

Recent studies also suggest that splicing-derived neoantigens may sometimes be public targets shared across patients and cancer types. T cell receptor (TCR) clones recognizing aberrant splicing neoantigens from GNAS and RPL22 have been identified, indicating that alternative splicing-derived targets may partly overcome intratumoral heterogeneity (8). D393-CD20 variants in B-cell lymphomas provide another strong example: the variant is translated in malignant B cells, and D393-CD20-specific CD4+ T cells can recognize lymphoma cells (26).

Serine/arginine-rich (SR) proteins and heterogeneous nuclear ribonucleoproteins (hnRNPs), which are the most significant mediators of splice-site recognition, are examples of splicing regulators that influence the activity of alternative splicing, which is mostly carried out by the spliceosome machinery (135).

Spliceosome inhibitors against SF3B1 (e.g. H3B-8800, E7107) have reached Phase I trials in myeloid malignancies including myelodysplastic syndromes (MDS), acute myeloid leukemia (AML) and chronic myelomonocytic leukemia (CMML). This gives proof of concept for pharmacological splicing modulation (136–138). Splice-switching antisense oligonucleotides (SSOs) redirect splicing to restore tumor-suppressive isoforms or induce immunogenic variants, and are advancing in pre-clinical and early clinical development (139, 140). RBM39 degraders (e.g. indisulam/E7070) have been evaluated in Phase II studies in solid tumors including lung, breast and colorectal cancers (141). To date, no splicing-targeted agent has been approved for oncology. Although the rationale is strong as splicing-derived neoantigens can improve T-cell recognition, combination strategies with checkpoint inhibitors are still mostly in the preclinical stage (142, 143).

The CD44 family illustrates how complex alternative splicing can be in tumor biology and target discovery. CD44s/CD44v isoforms are associated with cancer stem cell properties, EMT, invasion, metastasis and therapy resistance (27, 28). In lung cancer, the MEN1-CD44 axis shows crosstalk between splicing isoforms and tumor-suppressive mechanisms, as MEN1 may increase ferroptosis sensitivity by regulating CD44 alternative splicing (29). Exon 21-containing periostin variants in breast cancer also support the concept that extracellular matrix isoforms may function as diagnostic or therapeutic targets (30).

In hepatocellular carcinoma, deep RNA sequencing has identified differential skipped-exon events, only some of which overlap with differential gene expression (31). This suggests that gene-level analysis alone is insufficient for isoform-level changes. In glioma, the opposite biological effects of ITSN1-L and ITSN1-S isoforms show that different splice products from the same gene can influence tumor behavior in different directions (32). Overall, alternative splicing is not merely an additional neoantigen source. It shapes antigenic repertoire, phenotypic plasticity, invasion, microenvironmental interaction, and treatment sensitivity.

A recurring question is whether these tumor-specific splicing outcomes are driven by cis-regulatory RNA elements or by trans-acting RNA-processing factors. The two are best regarded as a single regulatory unit rather than as alternatives. Cis elements, including the 5′ and 3′ splice sites, the branch point, the polypyrimidine tract and exonic or intronic splicing enhancers and silencers, define where the spliceosome is able to act. Trans-acting factors, including SR proteins, hnRNPs and core spliceosomal components such as SF3B1, U2AF1, SRSF2 and ZRSR2, determine which of those permitted sites are actually used in a given cell. Tumors can therefore reach the same immunological endpoint from either direction. Somatic mutations that create a new cis element represent one route: systematic analysis of 8,656 TCGA tumors identified 1,964 splice-site-creating mutations, and the neoantigens generated by these events were predicted to be considerably more immunogenic than those arising from missense mutations, with a recurrent GATA3 event as a representative example (33). Change-of-function mutations in trans-acting factors represent the other route, because a single mutant spliceosomal protein such as SF3B1 shifts branch-point recognition and activates cryptic 3′ splice sites across hundreds of otherwise wild-type transcripts (34).

These two layers are interdependent, and the immunologically relevant output reflects both. A mutant trans factor mis-splices only those transcripts whose cis architecture is permissive, for example transcripts with weak polypyrimidine tracts, suboptimal branch points or atypical intron length. This dependency is why splicing-factor mutations generate reproducible, patient-shared neojunction sets rather than random ones, and why splicing-derived public neoantigens exist at all (8). It has also been exploited therapeutically: synthetic introns designed with cis elements that are efficiently spliced only in the presence of mutant SF3B1 enabled mutation-dependent expression of a suicide gene and selective killing of SF3B1-mutant leukemia, breast cancer, uveal melanoma and pancreatic cancer cells, while leaving isogenic wild-type cells unaffected (34). For target discovery the practical implication is that cis and trans information should be interpreted together: the trans-factor genotype predicts the class of aberrant events to expect, whereas the cis architecture of an individual transcript predicts whether a specific candidate junction will be produced reproducibly enough to justify further validation.

### 1.2 Alternative polyadenylation and 3′UTR remodeling in immune escape

The post-transcriptional regulation through which splicing and 3′UTR remodeling generate targetable antigens or immune escape programs are summarized in **Figure 2**. Alternative polyadenylation generates mRNA isoforms with distinct 3′ ends from the same gene locus. Even when the coding sequence is unchanged, this can strongly impact biology because 3′UTRs contain miRNA binding sites, RNA-binding protein motifs, AU-rich elements, localization signals and translational control sequences (19). The importance of alternative polyadenylation or APA in cancer has been underscored by widespread 3′UTR shortening in tumor cells (36, 144). Short 3′UTR isoforms can escape miRNA-mediated repression, increase protein production, and contribute to oncogene activation in the absence of genetic mutations (35–37).

**Figure 2:**
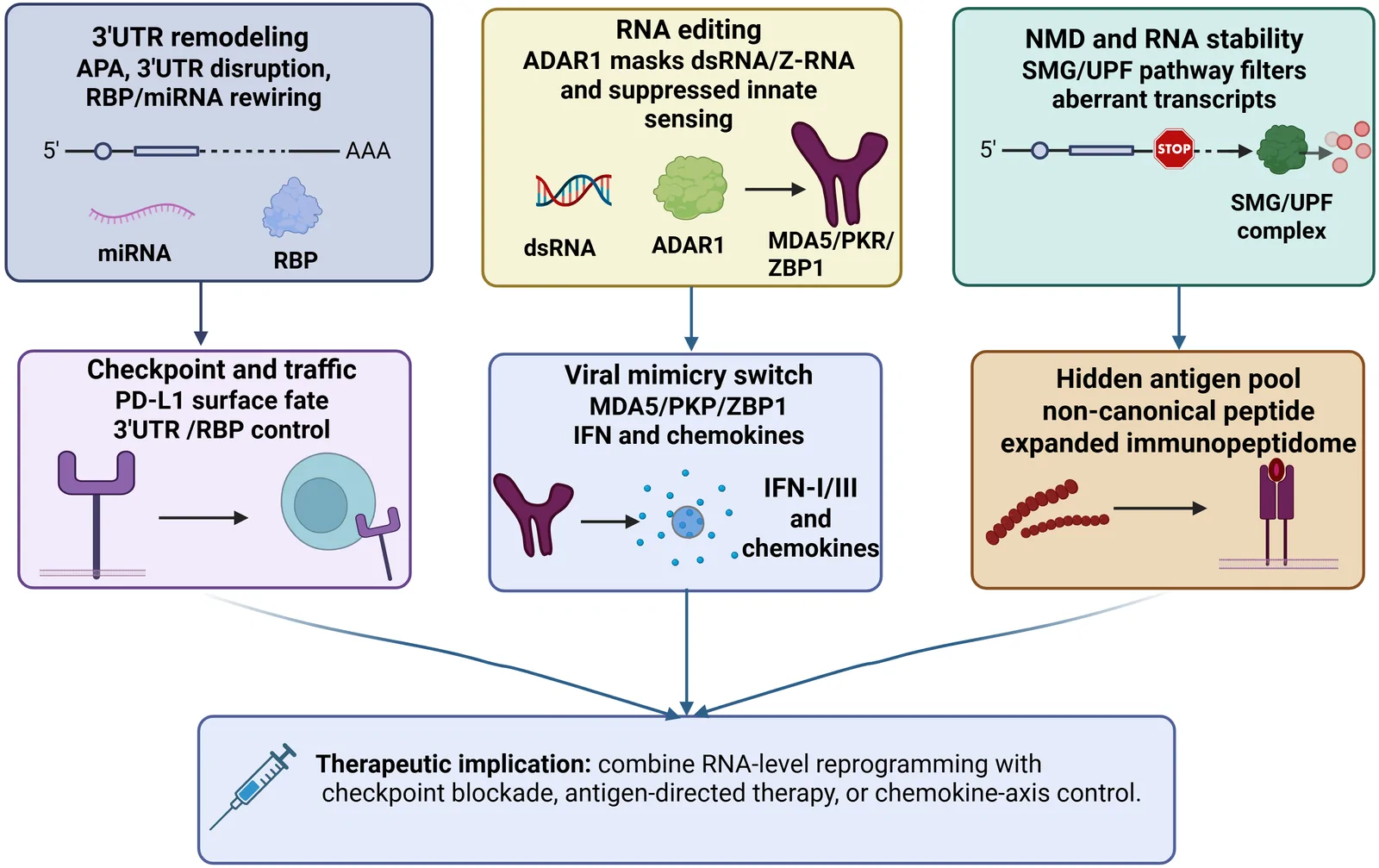
Immune checkpoints at RNA level generated by 3′UTR remodeling, RNA editing and NMD. Three major RNA-level regulatory axes defining tumor immune recognition and immunotherapy response are shown in this figure. First, post-transcriptional regulation of checkpoint expression, PD-L1 surface fate, and immune regulation may be altered by 3′UTR remodeling via alternative polyadenylation, 3′UTR disruption and RBP/miRNA rewiring. Second, ADAR1-mediated RNA editing can mask endogenous dsRNA or Z-RNA structures and preclude innate immune sensing via MDA5, PKR, and ZBP1 pathways. Loss or inhibition of this suppression may favor a viral mimicry-like state, interferon signaling and chemokine induction. Third, NMD and RNA stability pathways involving SMG/UPF factors may act as filters to remove aberrant transcripts and restrict the quantity of non-canonical antigenic peptides. Collectively, these mechanisms support the combination of reprogramming of RNA with checkpoint blockade, antigen-directed therapy or chemokine-axis modulation to improve antitumor immunity.

The same cis-trans logic applies to alternative polyadenylation. In cis, poly(A) site strength is determined by the AAUAAA hexamer, the upstream UGUA motif, the downstream GU/U-rich element and the surrounding RNA secondary structure. In trans, the relative abundance of the cleavage and polyadenylation machinery, including CPSF, CstF, CFIm and PABPN1, sets the threshold at which weak proximal sites become usable. Loss of the CFIm subunit CFIm25/NUDT21 illustrates how a trans change produces genome-wide but cis-dependent consequences: CFIm25 depletion shortened the 3′UTRs of at least 1,450 genes, increased the output of oncogenes including cyclin D1, and enhanced tumorigenicity in glioblastoma (43). Because proximal sites are preferentially selected only where the cis motifs are already marginal, the resulting 3′UTR shortening program is reproducible across tumors rather than stochastic, which is precisely what allows APA-derived signatures such as immune-landscape QTL and ImmAPA scores to function as biomarkers (38, 44). Neither layer is therefore sufficient alone. A 3′UTR event becomes immunologically meaningful only when a permissive cis architecture coincides with a trans environment that selects it, and the same principle applies to RNA editing, where ADAR1 abundance acts on dsRNA structures that are themselves specified in cis by inverted repeat elements.

APA-driven 3′UTR shortening of SPSB1 was shown to degrade MHC-I, enabling immune evasion in prostate cancer; a CRISPR/dCas13-based 3′UTR engineering platform (3′UTRCES) delivered via lipid nanoparticles reversed this and sensitized tumors to immune checkpoint blockade (ICB) in preclinical models (145).

One of the clearest immune-escape examples involves PD-L1/CD274. Structural variations that disrupt the 3′ region of PD-L1 can release the transcript from post-transcriptional repression, causing PD-L1 overexpression and immune escape (11). Therefore, PD-L1 immunohistochemistry alone may miss resistance mechanisms driven by 3′UTR disruption. In gastric cancer, 3′UTR variants have been associated with immune infiltration, TCR diversity and checkpoint inhibitor response. The predictive ability of a 3′UTR immune landscape Quantitative Trait Locus or QTL signature to outperform PD-L1 expression in some immune checkpoint inhibitors or ICI cohorts supports the biomarker potential of 3′UTR structure (38).

3′UTR remodeling can regulate PD-L1 beyond mRNA stability. DDX3 controls PD-L1 transport to the cell surface through binding to the PD-L1 3′UTR, and DDX3 loss or PD-L1 3′UTR truncation can cause intracellular PD-L1 accumulation (39). This shows that the 3′UTR may determine the cell-surface fate of immune checkpoint proteins, not only mRNA abundance. In this sense, 3′UTR remodeling can influence whether a checkpoint protein reaches the membrane where it can suppress T cells or remains intracellular and less available for immune synapse regulation. miRNA networks also contribute to this regulation. miR-155-5p suppresses PD-L1 mRNA and protein levels by targeting the PD-L1 3′UTR in lung adenocarcinoma cells (40). Similarly, miRNA-mediated regulation of the MICA/NKG2D axis indicates that NK-cell tumor surveillance may be modified post-transcriptionally (41). On the other hand, CSTF2 promotes APA-mediated destabilization of CXCL10 in pancreatic cancer and thus participates in suppression of T-cell infiltration; the inhibitor Forsythoside B reversed ICB resistance in preclinical models (146). NUDT21 facilitates 3′UTR extension of CDK19 in colorectal cancer connecting APA to immune escape via cholesterol (147). ImmAPA score across 31 cancer types predicts ICB response (44).

HLA-G 3′UTR variants provide a model for immune tolerance. Their association with post-transplant GVHD outcomes in acute leukemia supports the concept that 3′UTR genetics can influence immune outcomes (42). However, they are not always unidirectional. In some settings, 3′UTR truncation enables escape from miRNA repression; in others, lengthy 3′UTR isoforms may support protein localization and surface expression. The regulatory effects of long 3′UTRs in chemokine receptors such as CCR2 and CCR5 are therefore important to consider. Instead, the functional consequence depends on which regulatory motifs are gained or lost, which RBPs bind the transcript, and whether the final protein reaches the relevant cellular compartment. A simple and straightforward model in which short 3′UTRs are always protumoral and long 3′UTRs are always protective is unlikely to always be true.

### 1.3 Epitranscriptomic modifications: m6A and beyond

Epitranscriptomic modifications are chemical marks that reprogram RNA fate without changing RNA sequence. They can affect splicing, nuclear export, stability, subcellular localization, translation efficiency and degradation (20, 45, 46). m6A regulation involves writers, erasers and readers. METTL3, METTL14, WTAP, VIRMA, RBM15/15B and ZC3H13 deposit m6A marks; FTO and ALKBH5 remove them; and YTHDF1/2/3, YTHDC1/2, IGF2BP proteins and some hnRNPs interpret the marks to determine RNA fate (20, 47, 48).

METTL3 is the catalytic core of the m6A writer complex and installs the most abundant internal mRNA modification in eukaryotes. m6A is involved in almost all aspects of mRNA metabolism, including splicing, export, translation and decay (148–150). But METTL3 has strikingly context-dependent functions in cancer. It acts as an oncogene in AML, glioblastoma, hepatocellular carcinoma and colorectal cancer by stabilizing transcripts encoding c-MYC, BCL2, SOX2 and other oncoproteins (151–153). Contrarily, METTL3 functions as a tumor suppressor in triple negative breast cancer, where its downregulation promotes metastasis (183). This duality is indicative of the diversity of m6A-modified target transcripts and the downstream reader proteins that interpret the modification in each cellular milieu (153, 154).

The most advanced class of epitranscriptomic drugs are METTL3 inhibitors. STC-15, a first-in-class METTL3 inhibitor, is now in Phase Ib/II clinical trials in patients with advanced solid tumors and hematologic malignancies (155, 156). Preclinical studies demonstrated direct antitumor effects of STC-15 and its role in activating antitumor innate immune responses (184). STM2457 was effective in AML, neuroblastoma and gastric cancer xenograft models (157–159). EP652 was active in preclinical models of both liquid and solid tumors (160). METTL3 inhibition increases dsRNA levels, activating innate immune sensing and synergizing with checkpoint blockade in preclinical models (184). No m6A-targeting agent is currently approved (161).

Beyond individual m6A regulators, higher-resolution mapping technologies are now showing that tumors can reshape the m6A landscape in region-specific ways. Single-nucleotide m6A mapping by GLORI-seq has revealed distinct epitranscriptomic signatures in bladder cancer, including global methylation dilution and focal 3′UTR hypermethylation. This study shows that rather than being a straightforward worldwide gain or loss of methylation, cancer-associated m6A remodeling can be region-specific and functionally significant (54). The PD-L1 axis further shows the role of m6A in immune escape. In bladder cancer, PD-L1 mRNA stabilization is mediated by METTL3-induced m6A modification and IGF2BP1 recognition (55). Thus, 3′UTR structure and m6A marks can act on the same transcript to support immune escape. m6A reader proteins, especially the YTHDF family, also influence antigen presentation. YTHDF1 deficiency enhances the efficacy of anti-PD-L. YTHDF1 promotes translation of transcripts encoding lysosomal proteases in dendritic cells, thus reducing antigen cross-presentation (56). YTHDF1 has emerged as one of the most functionally relevant m6A reader proteins in cancer immunity. It can enhance the translation of m6A-marked transcripts through interactions with the translation machinery, although its translational impact may vary by context (57).

YTHDF1 is frequently elevated in human tumors and has been linked to poor outcome, with oncogenic roles reported in ovarian and bladder cancers through enhanced translation of proliferation-associated m6A-modified transcripts (58, 59). Beyond tumor-intrinsic effects, YTHDF1 also shapes the tumor microenvironment (TME). Its expression correlates with immune-cell infiltration and checkpoint gene expression in a cancer-type-dependent manner (60). In dendritic cells, YTHDF1 promotes translation of lysosomal cathepsins that degrade antigens, thereby limiting neoantigen presentation. Ythdf1 deletion enhances dendritic-cell-mediated antigen presentation, strengthens antigen-specific T-cell responses, slows tumor growth and improves anti-PD-L1 efficacy in mice (56). YTHDF1 has also been implicated in immune-mediated disease, such as celiac disease, where it promotes XPO1 expression and NF-κB-driven cytokine production after gluten-induced changes in the m6A methylome. In addition, YTHDF1 contributes to drug resistance, including cisplatin resistance in colorectal cancer through increased translation of GLS1; targeting YTHDF1 or GLS1 can restore drug sensitivity, and YTHDF1 knockdown also sensitizes colorectal cancer cells to fluorouracil and oxaliplatin. Together, these findings position YTHDF1 as a bridge between m6A-dependent translation, tumor growth, antigen presentation and therapy resistance (61, 62).

m6A erasers are also linked to immunotherapy resistance. FTO (Fat mass and obesity-associated protein) is associated with anti-PD-1 resistance in melanoma, whereas ALKBH5 may regulate anti-PD-1 response through lactate metabolism and accumulation of immunosuppressive cells (63, 64). Other RNA modifications are emerging. NAT10/ac4C inhibition can increase dsRNA accumulation, type I interferon response and CD8 T-cell activation, suggesting possible synergy with PD-1 blockade (65). Pseudouridine, m1A, m5C and m6Am may also affect RNA stability, translation and innate immune sensing (50, 66). The major epitranscriptomic axes with potential immunotherapy relevance are summarized in **Table 1**. These RNA marks should be interpreted in a cell-type-specific way, because the same pathway can support immune activation in antigen-presenting cells but promote immune escape in tumor cells or suppressive immune populations. Recent studies also suggest that abnormal nuclear RNAs can indirectly rewire RNA processing by forming aberrant ribonucleoprotein complexes. In a DUX4-driven model, stable intranuclear RNAs such as HSATII sequestered RNA methylation factors and formed HSATII–YBX1 complexes in an NSUN2-dependent manner, leading to altered RNA processing, including splicing. Although shown in FSHD, this mechanism is conceptually relevant to cancer because repeat-derived RNAs such as HSATII are often dysregulated in tumors and may connect RNA methylation, splicing, nuclear RNA organization and innate immune sensing (67).

**Table 1:** Epitranscriptomic modifications and their potential roles in cancer immunotherapy.

RNA modification/regulatory axis	Biomarkers	Effect on RNA	Consequence for cancer immunity	Relevance to immunotherapy	References
m6A writer system	METTL3, METTL14, WTAP, RBM15, VIRMA, ZC3H13	Deposits m6A marks on mRNA and influences stability, translation, decay and splicing	May alter interferon signaling, PD-L1 expression, antigen presentation and immune escape programs	Candidate therapeutic and biomarker axis for modulating anti-PD-1/PD-L1 response	(20, 47, 52)
m6A eraser system	FTO, ALKBH5	Removes m6A marks from RNA	May be linked to tumor metabolism, immunosuppressive microenvironments and resistance to checkpoint therapy	FTO or ALKBH5 inhibitors may have value in combination with immunotherapy	(63, 64)
m6A reader proteins	YTHDF1, YTHDF2, YTHDF3, YTHDC1/2, IGF2BP family	Recognize m6A-marked RNAs and regulate translation, decay or RNA stability	Can influence dendritic-cell antigen presentation, PD-L1 stability and T-cell responses	Reader proteins such as YTHDF1 may be targeted to enhance antigen presentation	(48, 56, 57)
METTL3/METTL14–IFN response axis	METTL3, METTL14, YTHDF2, STAT1, IRF1	Regulates the stability and degradation of transcripts involved in IFN-γ signaling	Can change how tumor cells respond to interferon signaling	In some models, METTL3/METTL14 suppression enhances anti-PD-1 response	(52)
m6A–PD-L1 axis	METTL3, IGF2BP1, PD-L1/CD274 mRNA	Regulates PD-L1 mRNA stability and translation	May increase PD-L1 expression and facilitate escape from T-cell cytotoxicity	Represents a post-transcriptional mechanism of resistance in checkpoint-refractory tumors	(55)
ac4C modification	NAT10	Regulates RNA stability and translation; linked to dsRNA and IFN responses	NAT10 activity may suppress antiviral-like immune signaling	NAT10 inhibition may enhance type I IFN signaling and CD8+ T-cell activity, potentially synergizing with PD-1 blockade	(65)
Pseudouridine	PUS family	Modulates RNA structure, stability, translation and immunogenicity	May affect endogenous dsRNA, retrotransposon responses and innate immune sensing	Emerging target space for inducing viral mimicry-like immune activation	(78, 79)
m1A	TRMT61A and related tRNA/mRNA regulators	Influences translational control	May be associated with macrophage STING–IFN-β signaling	Provides a potential regulatory layer for CAR-macrophage or myeloid-cell-based immunotherapies	(50, 66)
m5C/m6Am	NSUN family, TRDMT1, PCIF1	May affect RNA stability, translation, export and cap-adjacent regulation	Their links to cancer immunity are less well defined than m6A	Early-stage biomarker and therapeutic target candidates	(50, 77)

Technological validation is essential in this area. Databases such as m6AConquer can normalize data from different m6A profiling technologies and integrate m6A sites, transcriptomic changes and QTL information (68). However, m6A mapping alone is insufficient for target discovery and requires validation through RNA stability, translation, protein abundance, immunopeptidomics and functional immune assays. RNA modifications also sustain tumor growth by strengthening oncogenic RNA programs. In leukemia, glioblastoma and lung cancer, METTL3/METTL14 can deposit m6A marks on transcripts such as MYC, SOX2, EGFR and TAZ, increasing expression of genes that support proliferation and survival (69–71).

These m6A marks are interpreted by reader proteins in different ways: YTHDF1 can promote translation, whereas IGF2BP1–3 stabilize oncogenic mRNAs such as KRAS and CDK6 and can also support growth-promoting non-coding RNAs, including circNSUN2, circMDK and lncRNA ZFAS1 (72, 73). Yet m6A biology is not uniformly oncogenic. YTHDF2 can promote degradation of oncogenic transcripts such as EGFR, and the effects of FTO or ALKBH5 vary markedly across tumor types, acting as growth-promoting regulators in some contexts and showing more suppressive or complex roles in others (74–76). Similar principles extend beyond m6A. m5C, pseudouridine, A-to-I editing, ac4C, m1A and m7G can enhance oncogene expression, translational efficiency, ribosome biogenesis or suppression of tumor-inhibitory pathways through enzymes such as TET2, PUS1, TRUB1, DKC1, NAT10, TRMT6/61A and METTL1/WDR4, and these modification programmes intersect with established oncogenic signaling pathways across tumor types (49, 51, 77–80). Thus, RNA modifications should be viewed as active regulators of proliferative signaling and cell survival rather than passive marks on transcripts. Their therapeutic value in cancer immunotherapy will depend on whether these growth-promoting RNA programs can be targeted without impairing the immune-cell functions required for antitumor immunity.

Earlier studies showed that YTHDF1 promotes translation of m6A-containing mRNAs whereas YTHDF2 promotes their degradation, suggesting a model of division of labor (72). Subsequent work, however, showed that all three YTHDF paralogs are functionally redundant in promoting mRNA decay, challenging a role for YTHDF1 as a distinct translational enhancer (57, 162). Evidence that has emerged more recently suggests that each paralog has truly distinct functions, because of divergent N-terminal low-complexity domains, post-translational modifications, and subcellular localization (163). Crucially, YTHDF1-driven translation of ADAR1 mRNA following interferon stimulation links the m6A and A-to-I editing pathways, showing direct crosstalk between these modifications in innate immune regulation (164, 165).

### 1.4 RNA editing, innate immune sensing and resistance to immune therapy

RNA editing is a post-transcriptional process that results in a sequence of RNA that differs from the genomic DNA. The most common type of editing in mammalian cells is A-to-I editing by Adenosine Deaminase Acting on RNA or ADAR enzymes. A-to-I editing can be detected as an A-to-G-like change at the RNA level, since inosine is often read as guanosine in translation and sequencing (81). The relevance of RNA editing for immunotherapy stems from the processing of endogenous double-stranded RNA by tumor cells. Viral-like dsRNA structures can be formed by retroelements, inverted repeats, interferon-induced transcripts and misprocessed RNAs. MDA5, RIG-I, PKR and ZBP1 recognize these and induce interferon responses, inflammatory gene expression and cell death (12, 82). ADAR1 is central in this. Downregulation of the ADAR enzyme ADAR1 decreases A-to-I editing of IFN-induced RNA species, resulting in endogenous dsRNAs that are recognized by PKR and MDA5. This inhibits tumor growth and improves inflammatory responses in the tumor microenvironment. Importantly, ADAR1 loss was able to overcome PD-1 resistance in tumors with impaired antigen presentation (12).

ADAR1-mediated immune evasion is not only restricted to MDA5 and PKR. ADAR1 also regulates Z-form RNA levels and following ADAR1 loss, ZBP1 activation and RIPK3-mediated necroptosis can occur (83). Thus, ADAR1 functions as a complex innate immune checkpoint to limit the sensing of both A-form dsRNA and Z-RNA. Checkpoint ligands could also be regulated by RNA editing. ADAR editing of PVR/CD155 in colorectal cancer suggests that ADAR1 may have the potential to inhibit endogenous RNA alarm signals and modulate checkpoint ligand expression and T/NK-cell interactions (84).

Aicardi–Goutières syndrome is a severe autoinflammatory encephalopathy caused by loss-of-function mutations and driven by uncontrolled type I interferon signaling (166, 167). Paradoxically, this same immune‐suppressive function makes ADAR1 a therapeutic vulnerability in cancer. The tumors use ADAR1 to escape from dsRNA-induced antitumor immunity and ADAR1 inhibition can cause ZBP1-dependent inflammatory cell death (PANoptosis) resulting in tumor regression (83, 167). ADAR1 also promotes glioblastoma progression through an editing-independent RNA-binding mechanism by stabilizing CDK2 mRNA. Thus, ADAR1 is an immune guardian and a tumor accomplice depending on the circumstances (165). Direct ADAR1 inhibitors have not yet progressed to clinical trials, but CBL0137 (curaxin) that activates ZBP1-mediated necroptosis downstream of ADAR1, has reversed ICB unresponsiveness in melanoma models and has entered early-phase clinical evaluation (83). ATRA destabilizes ADAR1 protein preclinically and synergizes with PD-1 blockade in pancreatic cancer. ADAR1 deficiency sensitizes multiple preclinical models of pancreatic, lung and colorectal cancers to immunotherapy (168–170).

RNA editing is also linked to chemokine networks, which are important because they translate intracellular RNA sensing into altered cellular composition of the tumor microenvironment. The connection between RNA editing, innate immune sensing and chemokine-directed immune-cell trafficking is illustrated in **Figure 3**. When ADAR1 suppression is relieved or dsRNA sensing is activated, chemokines such as CXCL9, CXCL10, CXCL11, CCL5, CCL2, CXCL1 and CXCL5 are influenced by type I/II interferon, STAT1, IRF1 and NF-κB pathways (21, 85–89). Thus, RNA editing is not only a sequence-modifying process but also a regulator of innate sensing and immune cell trafficking. **Table 2** summarizes the key axes and therapeutic implications.

**Figure 3:**
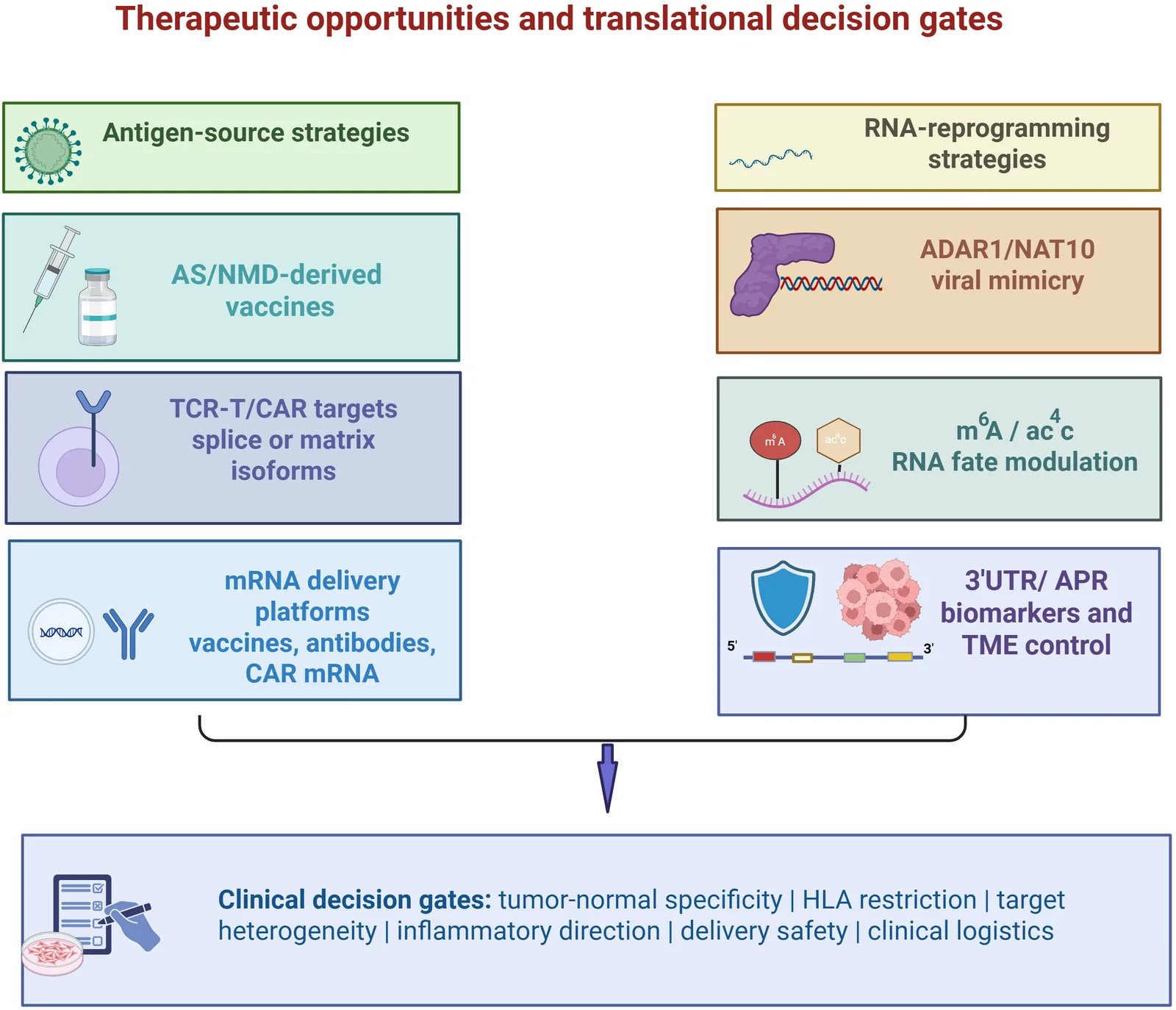
Therapeutic opportunities and translational decision gates for targets derived from mRNA processing. mRNA processing provides two complementary therapeutic opportunities in cancer immunotherapy. Antigen-source strategies are based on splicing- or NMD-derived tumor antigens and are applied in vaccine development, TCR-T/CAR-based targeting, and mRNA delivery platforms, such as mRNA vaccines, mRNA encoding antibodies, and mRNA-based CAR strategies. RNA-reprogramming strategies are being developed to modulate the ADAR1/NAT10-mediated viral mimicry pathway, m6A/ac4C-dependent RNA fate control, or 3′UTR/APA-associated biomarker or tumor microenvironment regulation to change tumor immune visibility. Several decision gates need to be translated from these strategies into the clinical setting such as tumor-normal specificity, HLA restriction, target heterogeneity, inflammatory direction, delivery safety and clinical logistics. These filters are critical for distinguishing biologically interesting RNA events from clinically actionable therapeutic targets.

**Table 2:** RNA editing, innate sensing and chemokine axes in immunotherapy resistance.

Axis	Biomarkers	Immunotherapy	Therapeutic implication	References
ADAR1–MDA5/PKR	ADAR1, dsRNA, MDA5, PKR	ADAR1 suppresses endogenous dsRNA sensing; ADAR1 loss can sensitize tumors to immunotherapy	ADAR1 inhibition combined with PD-1/PD-L1 blockade	(12, 82)
ADAR1–ZBP1	ADAR1, Z-RNA, ZBP1, RIPK3	ADAR1 prevents Z-RNA accumulation; ADAR1 loss may trigger necroptosis	Overcoming ICB resistance through ZBP1-mediated tumor-cell death	(83)
ADAR–checkpoint ligands	ADAR, PVR/CD155	RNA editing may alter immune-checkpoint ligand expression	Combination strategies involving TIGIT/PVR or PD-1 pathways	(84)
IFN–CXCL10/CXCR3	IFN-α/γ, STAT1, IRF1, CXCL10, CXCR3	Supports CD8+ T-cell recruitment and a “hot tumor” phenotype	Activation of RNA sensing pathways to increase T-cell infiltration	(21, 85)
CCL2/CCR2	CCL2, CCR2, monocytes, TAMs, Tregs	Promotes recruitment of immunosuppressive myeloid cells and may contribute to ICB resistance	CCR2 blockade combined with checkpoint inhibition	(21, 86, 87)
CCL5/CCR5	CCL5, CCR5, Tregs, MDSCs, TAMs, CD8+ T cells, NK cells	Can mediate either immune suppression or effector-cell homing depending on context	Cell-type-specific modulation of CCR5 signaling	(90, 91)
Dual CCR2/CCR5 targeting	BMS-813160, cenicriviroc, maraviroc	May reduce myeloid suppression and increase sensitivity to ICB	Combination strategies in PDAC, CRC, HCC, NSCLC and glioma	(88, 89)
ADAR1-high TAMs	ADAR1, GLI1 editing, SPP1, NF-κB	Associated with TAM polarization and therapy resistance	TAM-specific targeting of ADAR1 or RNA-editing programs	(12, 83)

The CCL2/CCR2 axis is critically involved in resistance to immunotherapy. CCR2 mediates monocyte and macrophage migration to gradients of CCL2, and these cells can suppress CD8+ T cell function by establishing tumor-associated macrophages (TAMs), myeloid-derived suppressor cells or MDSC- or Treg-favorable microenvironments (21, 86). CCL2/CCR2 plays a role in TAM recruitment, metastasis and immune escape in esophageal squamous cell and hepatocellular carcinoma or HCC (87, 88). The lactate-STAT3-CCL2 axis in pancreatic ductal adenocarcinoma connects metabolic resistance, TAM recruitment, and checkpoint resistance (89). CCL5/CCR5 signaling can recruit Tregs, MDSCs and TAMs, but may also promote CD8+ T-cell and NK-cell recruitment in some settings (90, 91). CCR5 therefore should be considered not as a universal suppressive target but as an immune-trafficking receptor with context-specific modulation. RNA editing-targeted strategies will depend on the ADAR1 suppression and the recruitment of CD8+ T cells or CCR2/CCR5-dependent immunosuppressive myeloid cells by this chemokine program.

### 1.5 NMD, RNA stability and antigen presentation

NMD is a highly conserved RNA surveillance pathway that detects mRNAs harboring premature termination codons and targets them for degradation. In cancer biology and immunotherapy NMD is more than quality control. It is a post-transcriptional filter that determines which transcripts are stable, which are translated, and which potential antigens are available to the immune system (9, 92–95).

The standard NMD model is based on a premature stop codon, upstream of the last exon-junction complex, but in human cells the decision is not a one-rule-fits-all. The efficiency of NMD can be affected by the position of the stop codon, the length of the 3′UTR, the structure of the exon, the half-life of mRNA, alternative splicing, and RNA-binding proteins. Lindeboom et al. analysis of 9,769 tumors revealed that NMD efficiency in human cancer follows multiple rules and that NMD-triggering mutations have selective consequences in tumor evolution (94). A major point for immunotherapy is that frameshift or nonsense mutation does not always lead to a novel antigen. If the mutant mRNA is degraded quickly by NMD, the abnormal protein or peptide may not be produced in sufficient amount. This is why NMD explains the gap between the genome based neoantigen prediction and the real, MHC-presented peptide repertoire (94). However, the relationship is complex because NMD-targeted mRNAs are not always silent. MHC-I peptides can be produced by premature translation-termination codon (PTC) containing mRNAs that are prevented from generating full-length proteins via non-canonical mechanisms, such as pioneer round translation (96).

A recent multi-omics study showed that reduced activity of the NMD kinase SMG1 predicts improved checkpoint inhibitor response. SMG1-targeted NMD inhibition stabilized premature termination codon-containing transcripts, many of non-mutational origin, reshaped the MHC-I immunopeptidome and increased neoantigen abundance to levels comparable with high-TMB tumors. Functionally, this enhanced antigen-dependent T-cell killing, activated tissue-resident T cells in patient-derived models and improved CPI efficacy *in vivo*, supporting NMD inhibition as a strategy to uncover otherwise inaccessible canonical and non-canonical neoantigens (9). SMG5/NMD inhibition reprograms the tumor microenvironment in colorectal cancer via the TRAF6–TBK1 axis and promotes CD8+ T-cell activation, revealing NMD as a filter for antigens and regulator of the immune-microenvironment (10).

In cancer, NMD has a clear two-sided role. Some tumors up-regulate NMD to degrade tumor-suppressor transcripts and eliminate immunogenic neoantigens, thereby limiting the immunogenicity of the tumor and promoting immune evasion (171, 172). Others suppress NMD to stabilize oncogenic transcripts that drive disease progression (173, 174). Beyond oncology, NMD contributes to embryonic development, stem cell differentiation, spermatogenesis, circadian rhythm and stress responses including the unfolded protein and integrated stress responses (175). This dual identity, quality-control guardian and context-dependent gene regulator, complicates therapeutic targeting: NMD inhibition may improve neoantigen presentation while paradoxically consolidating pro-tumorigenic transcripts (178). Nevertheless, NMD inhibition unmasks cryptic neoantigens and enhances antitumor immune responses in preclinical models, and both small-molecule inhibitors (NMDI-1, NMDI-14) and the approved hypomethylating agent 5-azacytidine, which inhibits NMD indirectly, have shown synergy with checkpoint inhibitors (178, 185, 186). Reduced NMD activity and enhanced sensitivity to NMD inhibition have been observed in tumors carrying SF3B1 or U2AF1 spliceosome mutations, suggesting a synthetic lethal strategy in MDS and related cancers (180). Therapeutic modulation of NMD remains preclinical, although repositioning of 5-azacytidine offers a near-term translational route (179, 186).

Therapeutic RNA platforms are also affected by RNA stability. Enhanced stability of antigen encoding mRNA in dendritic cells increases translation efficiency and T-cell stimulating capacity (97). The circRNA-based GPC3 vaccine can induce persistent antigen production and cDC1-CD8+ T-cell interaction, demonstrating that RNA stability is a direct therapeutic parameter for antigen presentation and T-cell activation (98). The same reasoning applies to synthetic mRNA vaccines, mRNA-encoded antibodies, and mRNA-CAR platforms, where UTR design, RNA stability and innate sensing determine both efficacy and tolerability. Since NMD governs many physiological transcripts, long-term systemic inhibition may lead to the buildup of defective transcripts or inflammatory stress in normal tissues. Therefore, tumor-specific or transient modulation of NMD and validation by immunopeptidomics is required (9, 95). This is especially true because NMD-sensitive RNAs may increase antigenicity in tumors, but they may also disrupt RNA homeostasis in normal tissues if the inhibition is widespread or prolonged. **Table 3** summarizes NMD-related mechanisms and implications in immunotherapy. The double function of NMD suggests that it is not only an antigen-suppressive mechanism but also regulates the balance between transcript quality, generation of abnormal peptides, inflammatory stress, and the real immunopeptidome.

**Table 3:** Nonsense-mediated decay, RNA stability and antigen presentation.

Mechanism	Tumor immunity	Potential contribution to immunotherapy	Key caution	References
NMD activation	Degrades PTC-containing or aberrantly processed transcripts	May reduce potential neoantigens and contribute to immune escape	it may protect cells from harmful truncated proteins	(92–94)
NMD inhibition	Stabilizes abnormal transcripts	May reveal hidden neoantigens and non-canonical peptides	Global inhibition may cause toxicity in normal tissues	(9)
SMG1 targeting	Affects a central kinase step in the NMD pathway	May reshape the immunopeptidome and improve checkpoint blockade response	Tumor-selective or transient targeting is needed	(9)
SMG5 targeting	Regulates later steps of the NMD pathway	May enhance CD8+ T-cell activation and ICB efficacy in CRC	Tumor context and cancer type are important	(10)
Pioneer round translation	Short antigenic peptides can arise from NMD-targeted RNAs	May unexpectedly contribute to the MHC-I peptide pool	RNA abundance and peptide presentation do not always correlate	(96)
RNA stability	Determines the lifespan and translation of antigen-encoding RNAs	Can improve the efficacy of mRNA or circRNA vaccines	Excessively prolonged or widespread antigen expression may create tolerance or toxicity risks	(97, 98)
Immunopeptidomics	Directly measures peptides presented on MHC molecules	Validates the clinical candidacy of NMD-derived targets	RNA-seq alone is insufficient	(17, 18, 25)
TME antigen presentation	Macrophage, DC and CD4/CD8 T-cell niches shape immune responses	Helps convert NMD-derived antigens into effective T-cell responses	Antigen production in tumor cells alone may not be enough	(56, 105)

### 1.6 Crosstalk and cooperation between mRNA-processing pathways

The preceding sections treat alternative splicing, APA, epitranscriptomic modification, RNA editing and NMD separately for clarity, but in tumor cells these are not independent modules. They act sequentially on the same transcript, and the immunological output usually reflects their combined action rather than any single step. Several well-defined couplings illustrate this point.

The clearest is alternative splicing coupled to NMD, or AS-NMD. Inclusion of an alternative exon carrying a premature termination codon, commonly termed a poison exon, converts a productive transcript into an NMD substrate, so that a splicing decision becomes a stability decision and ultimately determines whether any peptide is available for presentation. Poison exons are ultraconserved in the SR protein family and form a cross-regulatory network in which SR proteins control their own and each other’s abundance through AS-NMD. Poison-exon inclusion is differentially regulated in tumors relative to normal tissue, and splice-switching antisense oligonucleotides that reverse the increased skipping of the TRA2B poison exon detected in breast tumors alter breast cancer cell viability, proliferation and migration (99). The same coupling operates in prostate cancer, where FOXA1 controls the expression of trans-acting factors including SRSF1 and HNRNPK and thereby reduces the abundance of NMD-targeted isoforms, with inclusion of the NMD-determinant FLNA exon 30 predicting disease recurrence (100). From an immunotherapy standpoint this coupling explains an otherwise puzzling observation: many tumor-specific splice events are readily detectable at the RNA level yet contribute nothing to the immunopeptidome, because NMD removes them before translation is complete. It also explains why NMD inhibition unmasks non-canonical neoantigens that splicing analysis alone had already predicted (9, 10, 101).

RNA editing intersects the same axis. Because A-to-I editing alters the RNA sequence read by the splicing machinery, ADAR1 activity can directly determine splice-site usage and therefore NMD sensitivity. In SELENON, ADAR1-mediated editing of the U1 snRNA binding site at a 5′ splice site prevents exonization of an intronic Alu element; when this editing is reduced, the exonized transcript acquires a premature termination codon and is degraded by NMD (102). A single locus therefore links editing, splicing and decay. This has a direct implication for the strategies discussed in section 1.4: ADAR1 loss, the manipulation most often proposed to trigger a viral mimicry state, will simultaneously remodel the splicing and NMD landscape, with consequences for the antigen repertoire that are independent of its effect on dsRNA sensing.

Epitranscriptomic marks act as a further layer on top of both. The nuclear m6A reader YTHDC1 recruits SRSF3 and antagonizes SRSF10, placing m6A upstream of exon inclusion decisions (103). m6A deposition within 3′UTRs overlaps functionally with APA and with AU-rich element-mediated decay, as shown by focal 3′UTR hypermethylation in bladder cancer (54); the parallel cooperation between m6A sites and AU-rich elements in controlling cytokine transcript stability in activated CD8+ T cells is considered in section 1.9.2 (53). 3′UTR architecture closes the loop, because 3′UTR introns are common in TCGA tumors and their splicing alters NMD sensitivity, transcript abundance and oncogene networks (104). Aberrant nuclear RNAs can also rewire several of these steps at once by sequestering RNA methylation factors and altering downstream splicing (67).

The practical consequence is that these pathways should be profiled and modulated jointly rather than in isolation. A tumor-specific junction is only a candidate antigen if it survives NMD; an APA event matters only if the retained 3′UTR segment carries miRNA, RBP or m6A elements that are active in that cell type; and an intervention directed at one layer, such as ADAR1 or SMG1 inhibition, will predictably perturb the others. Combined profiling of junction-level splicing, NMD activity, editing index and modification maps on the same sample is therefore more informative than any single assay, and combination strategies that pair NMD or editing modulation with splicing-derived antigen targeting have a clear mechanistic rationale (101).

### 1.7 Technologies to identify immunotherapy targets based on mRNA processing

The investigations for immunotherapy targets derived from mRNA processing is more complex than classical DNA mutation-based neoantigen discovery. A target can be a splice junction, retained intron-derived peptide, APA-derived 3′UTR isoform, RNA-edited codon, NMD-escaping transcript or checkpoint mRNA stabilized by epitranscriptomic modification.

Bulk RNA-seq is often the first step. It can assess differential expression, fusions, splicing variants, RNA-level mutation evidence and some APA signals (14). However, it mixes signals from tumor, immune, stromal and endothelial cells and cannot define cell type or spatial context. Exon-level and isoform-level analyses are therefore important. Cancer-specific exon targets identified from 1,532 pediatric solid and brain tumor RNA-seq datasets were functionally validated by CAR-T experiments against EDB-FN1 and COL11A1 (13). Isoform peptides from RNA splicing for Immunotherapy target Screening (IRIS) can systematically prioritize alternative splicing-derived antigens for TCR and CAR-T cell targeting (7).

Short-read RNA-seq may not reconstruct full transcript structure reliably. Long-read sequencing and full-length transcriptomics are needed to define exon combinations, intron retention, and APA architecture (16). A study combining full-length transcriptomics with immunopeptidomics identified HLA-presented peptides and neoantigen-TCR pairs missed by classical approaches (17). Immunopeptidomics is critical because it directly measures naturally presented HLA/MHC peptides. Retained intron-derived neoepitope presentation on MHC-I supports the translation of RNA-level splicing events into real antigen presentation (25). Proteogenomic approaches can integrate genomic, transcriptomic and immunopeptidomic data to evaluate neoantigens, viral antigens, non-canonical antigens, and antigen-presentation defects (22).

Single-cell and spatial technologies identify in which cell type and tissue context a target is located. In HCC, using a combination of single-nucleus RNA-seq with spatial transcriptomics, identified CXCL12+ tumor-associated endothelial niches linked to immune resistance (105). Functional validation remains the final step. CRISPR knockout/activation screens can test whether ADAR1, METTL3, YTHDF1, SMG1, splicing factors or APA regulators affect antigen presentation and immunotherapy response (106). TCR/CAR-T cell co-cultures, organoid-immune systems, and *in vivo* models then determine true therapeutic value. These assays are necessary because computational prediction cannot establish whether a peptide is naturally presented at sufficient density or whether engineered immune cells can recognize the target without unacceptable off-tumor activity. These approaches should be considered a sequential validation framework rather than independent technologies, as summarized in **Table 4**. In practice, a target detected by RNA-seq should move through increasingly stringent filters: transcript structure, tumor-normal specificity, peptide presentation, cell-type context and functional immune recognition. This reduces the risk of selecting candidates that are abundant at the RNA level but absent from the HLA-presented peptide repertoire.

**Table 4:** Technologies for discovering mRNA-processing-derived immunotherapy targets.

Technology	What it provides	Use in mRNA-processing-derived target discovery	Reference
Bulk RNA-seq	Measures gene expression, fusions, RNA-level evidence for variants, splice junctions and some APA-related signals	First-line screening of tumor–normal expression differences, splice-junction usage and exon-level changes	(14, 132)
Exon-level expression analysis	Evaluates tumor specificity at the exon level rather than the whole-gene level	Identification of cancer-specific exons, surfaceome/matrisome targets and AS-derived CAR-T candidates	(13)
Isoform-level computational prioritization	Prioritizes tumor-associated or tumor-specific isoforms and splice-derived antigen candidates	Discovery of AS-derived targets for TCR-T, CAR-T or vaccine strategies	(7)
Long-read/HiFi sequencing	Resolves full-length transcript isoforms and complex RNA structures	Validation of exon combinations, intron retention, APA patterns and isoform architecture	(16)
Immunopeptidomics/Integrated transcriptomic–proteomic/immunopeptidomic workflows	Links complete transcript structures to naturally presented HLA-bound peptides Directly detects naturally presented MHC/HLA-bound peptides Combines RNA-level candidate discovery with peptide-level validation	Detection of neoantigens and neoantigen–TCR pairs missed by short-read RNA-seq or WES-based pipelines Validation of peptides derived from splicing, intron retention, NMD, non-canonical ORFs or RNA editing Reduces false-positive RNA-derived candidates by requiring evidence of antigen presentation	(17, 18)
Single-cell RNA-seq/single-nucleus RNA-seq	Defines which cell populations express candidate targets	Distinguishes tumor-cell, immune-cell, stromal, endothelial or fibroblast-derived signals	(14)
Spatial transcriptomics	Maps candidate expression within tumor tissue architecture	Evaluates immune exclusion, vascular barriers, T-cell proximity and ligand–receptor neighborhoods	(105)
CRISPR knockout/CRISPR activation screens	Tests whether candidate genes or RNA-processing regulators functionally affect immune phenotypes	Functional testing of ADAR1, METTL3, SMG1, YTHDF1, splicing factors or APA regulators	(106)
TCR/CAR-T functional validation	Tests whether candidate targets are recognized by engineered or endogenous T cells	IFN-γ release, killing assays, TCR reactivity, CAR-T activation and antigen-specific cytotoxicity	(13, 17)
AI/machine-learning-assisted prioritization	Uses interpretable or deep-learning models to refine antigen ranking by integrating MHC-I/MHC-II presentation, immunogenicity, TCR-recognition features, clonality and/or multi-modal sequence representations	Helps prioritize splice-derived, NMD-derived or other RNA-processing-derived peptide candidates before immunopeptidomics and functional T-cell validation	(133, 134)

### 1.8 Therapeutic opportunities

mRNA processing mechanisms generate biomarkers and create therapeutic intervention points. The studies can be considered at two levels: using tumor-specific products generated by alternative splicing, RNA editing, NMD, APA or epitranscriptomic regulation as target antigens, and reprogramming these mechanisms through pharmacological, genetic or RNA-based approaches (**Figure 4**). Major strategies and limitations are summarized in **Tables 5**, **6** provides a comparative overview of the level of biological evidence, current clinical development status, available therapeutic tools and principal translational challenge associated with each approach. Importantly, these strategies are not mutually exclusive. A tumor could be primed by ADAR1, NAT10 or NMD modulation, targeted with a splicing-derived vaccine or TCR-T cell product, and simultaneously protected from myeloid compensation through CCR2/CCR5-directed microenvironmental modulation.

**Figure 4:**
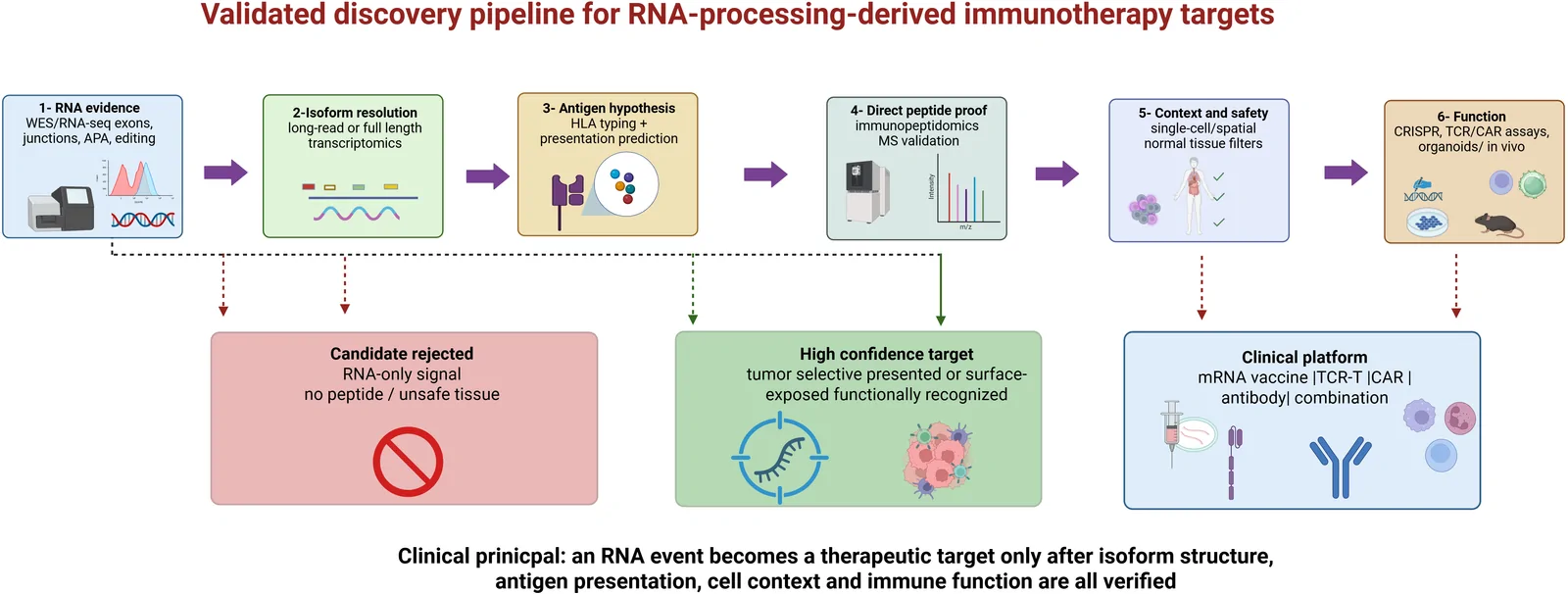
Validated discovery pipeline for immunotherapy targets based on RNA processing. This workflow describes a multi-layered approach for identification and validation of RNA processing-derived targets for cancer immunotherapy. WES/RNA-seq analysis to provide initial RNA evidence for exons, splice junctions, APA events, and RNA editing signals Candidate events are then refined by long-read or full-length transcriptomics to resolve isoform structure, followed by HLA typing and antigen presentation prediction to generate an antigen hypothesis. “Immunopeptidomics and mass spectrometry validation need direct peptide evidence. Single-cell, spatial, and normal tissue analyses are then utilized to define cellular context and safety, while CRISPR screens, TCR/CAR assays, organoids, and *in vivo* models are employed to assess functional relevance. RNA-only signals without peptide evidence or with unsafe tissue expression are rejected, whereas tumor-selective, presented or surface-exposed, and functionally recognized targets can advance to clinical platforms such as mRNA vaccines, TCR-T cells, CAR-based therapies, antibodies, or combination approaches.

**Table 5:** Therapeutic opportunities based on mRNA processing.

Therapeutic strategy	Targeted mechanism	Potential application	Main advantage	Major limitation	References
AS-derived neoantigen vaccines	Alternative splicing, neojunctions, intron retention	Personalized or off-the-shelf mRNA vaccines	Provides an additional target pool in low-TMB tumors	HLA presentation and T-cell recognition must be validated	(7, 8, 107)
TCR-T targets	Splice-derived peptides presented on HLA	TCR-T therapies against splicing-derived neoantigens	Can target intracellular protein-derived antigens	HLA restriction and cross-reactivity risk	(8, 17)
CAR-T/CAR-NK/CAR-M targets	Surface or matrix isoforms	Isoform targets such as CD44v6, EDB-FN1 and COL11A1	Enables HLA-independent targeting	Low-level expression in normal tissues may cause toxicity	(13, 28)
NMD inhibition	SMG1, SMG5, UPF/SMG pathway	Revealing hidden neoantigens; combination with ICB	May broaden the immunopeptidome	Global inhibition may cause toxicity and stress responses	(9, 10)
ADAR1/ZBP1 axis	RNA editing, dsRNA/Z-RNA sensing	Viral mimicry, IFN activation and reversal of checkpoint resistance	May be useful even in tumors with impaired antigen presentation	Self-RNA tolerance and autoinflammation risk	(12, 83)
m6A targeting	METTL3/METTL14, ALKBH5, FTO, YTHDF1	Regulation of IFN response, PD-L1, antigen presentation and TME programs	Offers druggable small-molecule targets	Strongly dependent on cell type and tumor context	(52, 55, 56, 63, 64)
NAT10/ac4C targeting	ac4C, dsRNA, IFN pathway	Combination with PD-1 blockade	May increase type I IFN signaling and CD8+ T-cell responses	Clinical evidence is still early	(65)
3′UTR/APA biomarkers	PD-L1 3′UTR, ilQTLs, miRNA/RBP motifs	Patient selection and prediction of ICI response	Provides information beyond PD-L1 IHC	Requires analytical standardization	(11, 38, 44)
mRNA therapeutic platforms	mRNA stability, UTR design, translation	mRNA vaccines, mRNA-encoded antibodies, mRNA-CAR-T/CAR-M	Rapid, modular and personalized	Delivery, immunogenicity and manufacturing logistics remain limiting	(97, 98, 109, 111)
CRISPR-Cas13/dCas13 RNA targeting	Transcript knockdown, dCas13 occupancy of splice-site cis elements, dCas13–ADAR programmable A-to-I editing	Depletion of immunosuppressive transcripts; induced splice or poison-exon events; multiplexed engineering of CAR T cells	Programmable, transient and reversible; acts on RNA without genomic double-strand breaks	Collateral bystander RNA cleavage, delivery and Cas protein immunogenicity	(119–124)
Splice-switching and gapmer antisense oligonucleotides	Cis elements: splice sites, branch points, ESE/ESS motifs, 3′UTR regulatory elements	Enforcing poison-exon inclusion; deliberate induction of shared neoantigens; 3′UTR-directed checkpoint or chemokine receptor modulation	Clinically validated chemistry with existing regulatory precedent	Biodistribution beyond liver and lymphoid tissue; repeat dosing required	(99, 101, 125)
Spliceosome inhibitors (SF3B1)	Core spliceosome; branch-point recognition	Splicing modulation in myeloid malignancies; generation of immunogenic isoforms	Clinical-stage chemistry; proof of concept for pharmacological splicing modulation	Phase I only; global splicing inhibition gives a narrow therapeutic window	(136–138)
RBM39 degraders	RBM39-dependent splicing	Solid tumors including lung, breast and colorectal cancer	Phase II experience already exists (indisulam/E7070)	Not developed as an immunotherapy; on-target splicing effects in normal tissue	(141)
METTL3 inhibitors	m6A writer complex; dsRNA formation and type I IFN	Advanced solid tumors and hematologic malignancies; combination with anti-PD-1	Most advanced epitranscriptomic drug class; STC-15 in Phase Ib/II	No approved agent; METTL3 is tumor-suppressive in TNBC, so direction of effect is context-dependent	(155, 156, 183, 184)
ADAR1-directed agents	ADAR1 protein stability; ZBP1-mediated cell death	Reversal of ICB unresponsiveness; combination with PD-1 blockade	Acts downstream or by degradation rather than by constitutive interferon suppression	No direct ADAR1 inhibitor in trials; Aicardi–Goutieres phenotype defines the autoinflammatory risk	(83, 166–168)
APA/3′UTR engineering	SPSB1 3′UTR shortening; MHC-I stability	Restoration of MHC-I in immune-cold tumors	Programmable and tumor-directed; converts cold tumors to ICT-responsive *in vivo*	Preclinical only; depends on lipid nanoparticle delivery	(145)
5-azacytidine repositioning	Indirect NMD inhibition	Unmasking cryptic neoantigens; combination with checkpoint blockade	Already approved in MDS, giving the shortest translational route in this class	Indirect mechanism; hypomethylating activity is not NMD-selective	(178, 186)

**Table 6:** Comparative overview of translational readiness across mRNA-processing–targeted approaches.

Approach	Level of biological evidence	Clinical development status	Available therapeutic tools	Major translational challenge
Splicing modulation (core spliceosome)	Strong. Splicing-factor mutations (SF3B1, U2AF1, SRSF2, ZRSR2) recurrently produce shared neojunctions; synthetic introns confer mutation-dependent killing (33, 34)	Phase I in myeloid malignancies (MDS, AML, CMML); no agent approved in oncology (136–138)	H3B-8800, E7107; RBM39 degraders (indisulam/E7070) in Phase II solid tumors (141)	Global spliceosome inhibition affects normal splicing; narrow therapeutic window and dose-limiting toxicity (105)
Splice-switching antisense oligonucleotides	Strong in principle. Poison-exon and cryptic-exon switching validated preclinically, including TRA2B in breast tumors (99)	Preclinical to early clinical (139, 140)	Splice-switching ASOs (steric block); gapmer ASOs (RNase H1-mediated degradation) (125)	Biodistribution beyond liver and lymphoid tissue; effect is transient by design and requires repeat dosing
APA/3′UTR engineering	Strong mechanistic evidence. SPSB1 3′UTR shortening degrades MHC-I; CFIm25/NUDT21 loss shortens ≥1,450 genes (43, 145)	Preclinical only (145)	LNP-delivered 3′UTRCES (CRISPR/dCas13); miRNA/ARE/RBP-motif-guided 3′UTR design (145)	*In vivo* delivery; no clinical agent exists; requires tumor-selective targeting
m6A writers (METTL3)	Strong but context-dependent. Oncogenic in AML, glioblastoma, HCC and colorectal cancer; tumor-suppressive in TNBC where loss promotes metastasis (151, 152, 183)	Phase Ib/II (STC-15) in advanced solid tumors and hematologic malignancies; no m6A-targeting agent approved (155, 156, 161)	STC-15 (oral, first-in-class); STM2457; EP652 (157–160)	m6A is the most abundant internal mRNA mark and acts in every cell type; opposite direction of effect between tumor types complicates patient selection (183, 184)
m6A erasers and readers (FTO, ALKBH5, YTHDF1)	Moderate. FTO linked to anti-PD-1 resistance in melanoma; YTHDF1 controls dendritic-cell cross-presentation (56, 63, 64)	Preclinical (56, 63, 64)	Tool compounds; genetic depletion; no clinical-stage selective inhibitor	Reader paralogue redundancy remains contested; on-target effects in normal immune cells (162, 163)
NAT10/ac4C	Emerging. NAT10 inhibition raises dsRNA, type I interferon and CD8+ T-cell activation (65)	Preclinical only (65)	Tool inhibitors; no clinical-stage agent	Thinnest evidence base of the targets discussed; ac4C also modifies rRNA and tRNA, so inhibition is not mRNA-selective
ADAR1/RNA editing	Strong. ADAR1 loss sensitizes tumors to immunotherapy via MDA5/PKR; editing-independent roles also described (12, 165)	No direct ADAR1 inhibitor in trials. CBL0137, acting downstream via ZBP1, is in early-phase evaluation (83)	CBL0137 (curaxin); ATRA (destabilizes ADAR1 via USP7); genetic depletion (83, 168)	ADAR1 loss-of-function causes Aicardi–Goutieres syndrome through uncontrolled type I interferon — human genetic evidence of autoinflammatory risk from systemic inhibition (166, 167)
NMD inhibition	Strong. NMD degrades PTC-containing neoantigen transcripts; SF3B1/U2AF1-mutant cells show selective NMD dependency (171, 172, 180)	Preclinical; 5-azacytidine offers a repositioning route as an approved agent in MDS (178, 186)	SMG1 inhibitors; NMDI-1 and NMDI-14 derivatives; 5-azacytidine (178, 185, 186)	May unmask neoantigens while simultaneously stabilizing pro-tumorigenic transcripts; NMD has essential physiological roles in development, stem-cell differentiation and stress responses (171, 175–178)
AS-derived neoantigen vaccines	Most mature antigen class. Off-the-shelf AS-derived mRNA vaccination in HCC; public splicing neoantigens support scalable targeting (8, 107)	Preclinical to early clinical (8, 107)	mRNA vaccine platforms; shared/public neojunction target sets	Requires confirmation that the RNA-level event reaches the HLA-presented peptide repertoire; HLA restriction limits population coverage (1, 22)
TCR-T and CAR-based targeting of RNA-derived antigens	Validated for defined targets. CAR-T against cancer-specific exons EDB-FN1 and COL11A1 in pediatric tumors (13)	Preclinical to early clinical (13, 17)	TCR-T; CAR-T, CAR-NK, CAR-macrophage; antibody/ADCC against surface or matrix isoforms	Isoform-level specificity against normal tissue; antigen heterogeneity and escape
Cas13/dCas13 RNA targeting	Strong proof of concept. dCasRx redirects splicing; dCas13–ADAR2 (REPAIR) enables programmable editing; multiplexed Cas13d improves CAR T fitness (119, 120, 122)	Preclinical only (119–124)	Cas13d; catalytically inactive dCas13 fusions (RS, hnRNP, ADAR2); chemically induced dCas13 splice modulation (121)	Collateral, sequence-independent cleavage of bystander RNAs; delivery and Cas immunogenicity for systemic use (123, 124)

Alternative splicing (AS)-derived neoantigens are among the most mature targets. They can be used for mRNA vaccines, TCR-T cells, and immunomonitoring. AS-derived, off-the-shelf mRNA vaccination in HCC suggests that these targets may provide broader patient coverage (8, 107). Public splicing neoantigens further suggest scalable targeting beyond personalized vaccines (8). Surface or matrix isoforms can also be used for CAR-T cell, CAR-NK cell, CAR-macrophages, antibody, or ADCC approaches. CAR-T cell validation of cancer-specific exon targets, such as EDB-FN1 and COL11A1 in pediatric tumors, demonstrates the translational potential of this approach (13).

Reprogramming RNA processing is a second therapeutic opportunity. NMD inhibition may reveal hidden neoantigens (9, 10). ADAR1 targeting can activate dsRNA/Z-RNA sensing and push tumors toward a viral mimicry-like state (12, 81–83, 85). Splicing modulation can alter tumor-dependent isoforms through splice-switching antisense oligonucleotides or selective RNA therapies, although global spliceosome inhibition requires caution because of toxicity.

Epitranscriptomic regulators are also promising combination targets. METTL3, METTL14, ALKBH5, FTO, YTHDF1 and NAT10 can influence interferon response, PD-L1 expression, antigen presentation, and the tumor microenvironment (52, 55, 56, 63–65). Evaluation of the oral METTL3 inhibitor STC-15 suggests that this field is moving toward druggable targets.

APA and 3′UTR remodeling are important as biomarkers and therapeutic intervention points. Because 3′UTRs contain miRNA sites, AU-rich elements, RBP motifs, localization signals and translational control sequences, two mRNA isoforms with the same coding sequence may differ in stability, translation, intracellular distribution and protein surface localization (11, 19, 35–39). PD-L1 3′UTR disruption can release the transcript from repression and contribute to overexpression and immune escape; CD274 3′UTR integrity may therefore explain resistance not captured by PD-L1 IHC alone (11). Gastric cancer 3′UTR variants associated with immune infiltration, TCR diversity and ICI response support their role in patient selection (38). The pan-cancer ImmAPA approach also identified immune-related APA events associated with prognosis, immune escape and ICB biomarkers (44).

3′UTR-RBP interactions offer a mechanistic and potentially targetable layer. In the DDX3–PD-L1 3′UTR axis, DDX3 directs AP2 through the CD274 3′UTR to facilitate surface transport of newly synthesized PD-L1; DDX3 loss or PD-L1 3′UTR truncation can reduce surface checkpoint density (39). This suggests that disrupting RBP-3′UTR interactions may target checkpoint production and trafficking rather than the checkpoint protein itself.

Translating these opportunities will depend on defining who should receive them and on what evidence. For NMD-directed strategies, spliceosome genotype offers the clearest selection criterion, since SF3B1- and U2AF1-mutant tumors show selective dependency on NMD (180). For ADAR1-directed approaches, editing index, interferon-stimulated gene signatures and, in light of T-cell-intrinsic ADAR1 activity, editing status within tumor-infiltrating lymphocytes are candidate stratifiers (12, 182). For splicing- and APA-derived antigens, HLA genotype determines who can present a given target at all, so allele coverage becomes an enrollment parameter rather than a *post hoc* explanation of non-response (1, 22). Biomarker maturity varies considerably across these approaches. The pan-cancer ImmAPA score and 3′UTR integrity measures are exploratory but already prognostic in retrospective cohorts (44), whereas immunopeptidomic confirmation that a candidate junction reaches the HLA-presented repertoire remains the only direct evidence that an RNA-level event is immunologically actionable, and is not yet routine (17, 22). Conventional biomarkers are unlikely to substitute, because tumor mutational burden, microsatellite instability and PD-L1 immunohistochemistry do not capture isoform identity, 3′UTR integrity or editing activity.

This logic extends to chemokine receptors. The long CCR2 3′UTR suppresses reporter expression in primary human CD4+ T cells and macrophages, RBP binding sites regulate this region, and CRISPR-Cas9 knockout of the CCR2 3′UTR increases CCR2 mRNA and protein levels (108). Deletion of the CCR2 3′UTR also doubles CCR2 mRNA half-life and nuclear/cytoplasmic transcript accumulation (108). Although not a cancer study, this is relevant because the CCL2/CCR2 axis contributes to immunotherapy resistance through TAM, monocyte, and Treg trafficking. The finding suggests that chemokine receptor signaling can be regulated not only through ligand abundance or receptor blockade, but also through 3′UTR-RBP control of receptor mRNA stability and localization.

3′UTR remodeling should not be limited to APA. A recent study using The Cancer Genome Atlas samples showed that 3′UTR introns are common and that 3′UTR splicing can affect NMD sensitivity, transcript expression and oncogene networks (104). Thus, 3′UTR architecture connects APA, splicing, NMD, RNA stability and translation. Therapeutically, this supports using 3′UTR/APA profiles as ICI biomarkers, targeting DDX3-CD274 or hnRNPA0-CCR2-like interactions, and optimizing synthetic 3′UTRs in mRNA vaccines, and mRNA-CAR T cell platforms (44, 104, 108–113).

RNA processing-targeted therapies can also be combined with chemokine-axis modulation. ADAR1, NAT10, or NMD targeting may increase interferon and chemokine responses, but if CCL2/CCR2 or CCL5/CCR5 dominates, immunosuppressive monocytes, TAMs, MDSCs, or Tregs may also be recruited. CCR2/CCR5 targeting can therefore be combined rationally with RNA processing-based immune activation (21, 86–89).

mRNA processing is also increasingly a design parameter for RNA therapeutics themselves, rather than only a target within the tumor. Because 5′ and 3′UTR architecture, nucleoside modification and transcript stability determine how long an antigen is produced and how strongly innate sensors are engaged, the processing rules discussed above govern the potency of mRNA and circRNA vaccines, mRNA-encoded antibodies and mRNA-based *in vivo* CAR engineering (97, 98, 109–118). Splicing-derived antigens can be encoded directly in these platforms, as shown by off-the-shelf alternative splicing-derived mRNA vaccination in hepatocellular carcinoma (107), and synthetic 3′UTRs can be optimized using the same miRNA, AU-rich element and RBP motif logic that tumors exploit for immune escape (44, 104, 108). These platforms are considered here only insofar as mRNA-processing biology informs their design; delivery chemistry and cell-engineering aspects fall outside the scope of this review.

#### 1.8.1 Programmable RNA-targeting tools: CRISPR-Cas13 systems and antisense oligonucleotides

Reprogramming mRNA processing requires tools that act on RNA rather than DNA, so that the genome is left intact and the effect is titratable and reversible. Two classes of programmable RNA-targeting technology are now sufficiently mature to be considered for immunotherapy applications.

RNA-targeting CRISPR systems. Type VI CRISPR effectors (Cas13) are RNA-guided ribonucleases that bind and cleave RNA without DNase activity, giving transient, dose-dependent knockdown without genomic double-strand breaks. Catalytically inactive Cas13, or dCas13, converts the same platform into a programmable RNA-binding module that can be directed to cis-regulatory elements of a pre-mRNA. Konermann et al. targeted virally delivered, catalytically inactive CasRx, a compact Cas13d ortholog, to cis elements of pre-mRNA in order to manipulate alternative splicing and correct a dysregulated isoform ratio in a neuronal disease model, establishing that splice-site choice can be reprogrammed by an RNA-guided protein alone (119). Fusing effector domains to dCas13 extends this principle. dCas13-ADAR2 fusions (REPAIR) direct programmable A-to-I editing to selected transcripts without strict sequence constraints (120), and dCas13 fused to RS or hnRNP domains functions as an artificial splicing factor whose activity is dependent on binding position relative to the regulated exon, an approach used to map the elements controlling the oncogenic TRA2B poison exon (99). Chemically induced dCas13 platforms have more recently tethered a splicing-modulator small molecule to a catalytically inactive RfxCas13d, achieving locus-restricted exon inclusion at ligand doses far below those required for the free compound, which begins to address the specificity limitations of systemic splicing modulators (121).

Three applications follow for cancer immunotherapy. First, Cas13 can deplete an immunosuppressive transcript, such as an m6A regulator, a checkpoint mRNA or an NMD factor, in a transcript-selective and potentially cell-type-restricted manner that a small-molecule inhibitor cannot achieve, and without creating a permanent genomic lesion. Second, dCas13-directed splice modulation provides a route to induce a defined tumor-specific junction or poison-exon event on demand, converting mRNA processing from a discovery substrate into a controllable antigen-generating mechanism (101). Third, Cas13 can be applied to the effector cell rather than to the tumor: multiplexed Cas13d effector guide arrays enabled quantitative, reversible and combinatorial knockdown in primary human T cells without targeting genomic DNA, suppressed inhibitory receptor upregulation in a model of CAR T cell exhaustion, and improved CAR T cell fitness and antitumor activity *in vitro* and *in vivo* (122). Two caveats are important. Cas13 exhibits collateral, sequence-independent cleavage of bystander RNAs after target engagement; whether this occurs in mammalian cells was initially disputed, but it is now documented for abundant and repeat-containing targets, where it depletes endogenous transcripts, interferes with cellular processes and activates stress and apoptotic responses, and mitigation requires expression-level control such as negative autoregulation (123, 124). Delivery and Cas protein immunogenicity also remain unresolved for systemic administration.

Antisense oligonucleotides. ASOs are the clinically most advanced means of redirecting mRNA processing. Splice-switching ASOs bind a cis element, such as a splice site, branch point or splicing enhancer or silencer, without recruiting RNase H, and thereby sterically block or promote use of that site and shift isoform ratios. Gapmer ASOs, in contrast, recruit RNase H1 to degrade the target transcript. Approved splice-switching ASOs in neuromuscular disease have established the chemistry, pharmacology and regulatory framework for this modality, and several RNA-targeting agents including ASOs are now in oncology trials (125). Three uses are directly relevant here. First, ASOs can enforce an immunologically favorable isoform, as demonstrated by splice-switching ASOs that reverse the tumor-associated skipping of the TRA2B poison exon; the same principle applies to any poison exon whose inclusion redirects a transcript into NMD (99). Second, ASOs can be used deliberately to generate neoantigens by forcing inclusion of a cryptic or poison exon that produces a frameshifted, tumor-restricted peptide. This induced-neoantigen strategy is attractive because the resulting epitope is shared by design rather than private to one patient, and because it can be rationally combined with NMD inhibition so that the aberrant transcript is translated rather than degraded (101). Third, ASOs directed at 3′UTR regulatory elements can modulate checkpoint or chemokine receptor output without altering the coding sequence, which is the logical therapeutic extension of the DDX3-CD274 and hnRNPA0-CCR2 3′UTR mechanisms discussed above (39, 108).

The limitations of both platforms are those common to RNA therapeutics: biodistribution beyond liver and lymphoid tissue, the need for repeat dosing because the effect is transient by design, and the requirement to demonstrate that the intended RNA-level change is actually reflected in the HLA-presented peptide repertoire. Neither platform removes the validation chain described in section 1.7; they change what can be manipulated, not what must be proven.

### 1.9 Challenges and unresolved questions

Although mRNA processing-derived targets offer broad opportunities, clinical translation remains challenging. A change at the RNA level is not automatically a therapeutic target. A splice variant, 3′UTR alteration, RNA editing event, or NMD-escaping transcript must be present at the RNA level, expressed sufficiently, reflected at the protein or peptide level, presented on HLA/MHC, safe in normal tissues, and able to induce a functional T-cell response (17, 18). Key unresolved questions and possible solutions are summarized in **Table 7**. These questions are especially important for high-potency modalities such as TCR-T and CAR-T cells, where a low level of normal tissue expression or an unrecognized cross-reactive peptide may create clinically meaningful toxicity.

**Table 7:** Unresolved questions and possible solutions.

Major challenge	Key question to answer	Why it matters	Possible solution	References
RNA–peptide mismatch	Does the RNA-level event become an HLA-presented peptide?	Reduces false-positive target selection	Immunopeptidomics, ribosome profiling, proteomics	(17, 18, 22)
Normal tissue safety	Is the target isoform expressed in critical normal tissues?	Prevents CAR/TCR-related toxicity	Single-cell normal atlases, proteomics, normal tissue-panel validation	(13)
Tumor heterogeneity	Is the target preserved across tumor clones?	Determines antigen-loss and relapse risk	Multi-region sequencing, spatial transcriptomics	(8, 24)
HLA restriction	In how many patients can the target be presented?	Determines patient coverage	HLA population analysis, multi-peptide vaccine design	(129, 130)
Antigen-presentation defects	Does the tumor retain the HLA/MHC pathway?	Required for TCR- or vaccine-based responses	B2M, HLA, TAP and IFN-pathway analysis	(1, 22)
Context-dependent effects	Does the same RNA-processing factor act similarly in tumor and immune cells?	Global inhibition may create toxicity	Cell-type-specific analysis and targeted delivery	(12, 20, 56)
Direction of inflammation	Does the therapy recruit CD8+ T cells or TAMs/MDSCs?	Viral mimicry alone may not be sufficient	Chemokine profiling, combination strategies	(21, 86, 89)
Technological standardization	Which platform is reliable enough for clinical decision-making?	Enables comparability across studies	Standardized pipelines, quality control and prospective validation	(14, 68, 132)
Clinical logistics	How quickly can a personalized target be manufactured?	Time is limiting in aggressive tumors	Automated bioinformatics and rapid mRNA manufacturing	(22, 128)
Combination design	Which patients should receive ICI, vaccine, ADAR/NMD/m6A targeting and chemokine blockade together?	Needed to improve efficacy while limiting toxicity	Biomarker-guided adaptive clinical trials	(9, 38, 44)

One major problem is RNA-protein-peptide mismatch: a splicing event or NMD-sensitive transcript detected by RNA-seq may never reach the HLA-presented peptide repertoire, so transcriptomics-only target selection carries a high false-positive risk. The antigen-processing steps that determine this are considered in section 1.9.1. A second challenge is tumor-normal specificity, which is essential for any therapy intended to redirect potent T-cell or myeloid-cell activity. Even if an isoform appears highly expressed in tumor, it may occur at low levels in normal tissues, developmental tissues, activated immune cells or stressed epithelium. For CAR-T and TCR-T cell therapies, even low-level normal expression can create toxicity (13).

Tumor heterogeneity and target loss are also important. mRNA processing events may vary between clones, and treatment pressure may eliminate the target isoform or impair antigen presentation. Public splicing neoantigens may partially reduce this problem, but intratumoral conservation, HLA restriction and stability under treatment pressure must be validated for each target (8).

Context-dependent effects of RNA processing factors add further complexity. METTL3, METTL14, ALKBH5, FTO, YTHDF1, ADAR1, NAT10, SMG1, and splicing factors may have different effects in tumor and immune cells (12, 20, 56). Widespread blockade of critical immune checkpoints may therefore cause toxicity, autoinflammation, or immune imbalance. The direction of inflammation is another critical question. RNA editing, NMD, or NAT10 targeting may increase interferon responses, but whether this recruits CD8+ T cells through CXCL10 or TAM/MDSC/Treg cells through CCL2/CCR2 or CCL5/CCR5 may determine treatment success (21, 86–89, 91).

Technological standardization is unresolved. Short-read RNA-seq, long-read nucleic acid sequencing, immunopeptidomics, single-cell/spatial omics, and artificial intelligence tools measure different biological layers. Clinical target selection requires standardized, reproducible, and prospectively validated integration (14–18, 22, 106, 126–131). Classical biomarkers may be insufficient for this class of therapies. Tumor mutational burden, microsatellite instability or PD-L1 immunohistochemistry alone do not capture isoform specificity and HLA presentation for splicing-derived targets, 3′UTR integrity and ilQTL signatures for APA/3′UTR remodeling, editing index and dsRNA sensing for ADAR1, SMG1/SMG5 activity and immunopeptidome changes for NMD, or modification maps and reader/eraser context for m6A/ac4C (9, 38, 68).

#### 1.9.1 Antigen processing, presentation and HLA restriction

The immunological relevance of RNA processing-derived targets critically depends on whether aberrant transcripts are translated, processed by the proteasome, transported by TAP into the endoplasmic reticulum and loaded onto HLA/MHC-I molecules at a sufficient density to engage cognate T-cell receptors (1, 22). Splicing-derived neojunction peptides and NMD-escaping transcripts have to pass through all steps of this antigen-presentation cascade. If any step of the process fails, the event at the RNA level is immunologically silent. Immunopeptidome studies have validated presentation of peptides derived from retained introns on MHC-I, however the efficiency depends on the HLA allotype, the preferences of proteasomal cleavages and the affinity of TAP-binding (17, 25). Commonly observed in immunotherapy-resistant tumors, frequent loss of beta-2 microglobulin (B2M), downregulation of HLA class I, and TAP deficiency all result in the abolition of T-cell recognition regardless of the abundance of neoantigen (1). Thus, strategies directed to RNA processing have to consider the integrity of antigen presentation as a prerequisite for efficacy. An important limitation is HLA restriction that limits population applicability. A neoantigen restricted to a single common HLA allotype will be presentable in only a subset of patients, and allele frequencies differ substantially between populations, thus necessitating multi-peptide vaccine designs or selection of HLA-diverse targets to achieve broad patient coverage (1, 22). Regulation of antigen presentation at the RNA level involves epitranscriptomic modifications as well. In dendritic cells, METTL3-dependent m6A methylation of transcripts encoding lysosomal cathepsins boosts their translation after sensing by YTHDF1, which speeds up antigen degradation and reduces the pool of peptides for MHC-I cross-presentation (56, 181). Deletion of YTHDF1 in dendritic cells maintains antigen integrity, improves cross-presentation, enhances antigen-specific CD8+ T cell responses and increases anti-PD-L1 efficacy in preclinical models (56).

#### 1.9.2 RNA processing in T-cell dysfunction and exhaustion

New evidence suggests that RNA processing mechanisms are active not only in tumor cells but also in tumor-infiltrating T cells to promote immune dysfunction. Single-cell RNA editing profiling in colorectal cancer has demonstrated elevated ADAR1 activity only in tumor-infiltrating T cells, which has been associated with an exhausted and proliferative T-cell state with compromised cytotoxic function (182). Functional experiments confirmed that ADAR1 overexpression in T cells promotes exhaustion through activation of the TGF-β–SMAD signaling pathway and *in vivo* adoptive transfer models demonstrated that T-cell-intrinsic ADAR1 limits antitumor efficacy (182). In the clinic, high ADAR1 expression in T cells correlated with reduced anti-PD-1 response across multiple immunotherapy cohorts, suggesting that T-cell ADAR1 may serve as a predictive biomarker and therapeutic target distinct from tumor-intrinsic ADAR1 functions (182). m6A modifications also regulate T cell effector functions. In activated CD8+ T cells, m6A sites in proximity to AU-rich elements in 3′UTRs label meta-unstable immunoregulatory mRNAs, such as TNF and other cytokine transcripts, for rapid decay in response to activation by the activity of YTHDF proteins (53, 54). This m6A–AU-rich element (ARE) crosstalk regulates cytokine output during effector responses and could serve as an adjustable axis for enhancing T cell activity during immunotherapy. Moreover, the m6A modification by METTL3/METTL14 might also regulate the IFN-γ signaling transcripts via YTHDF2-dependent decay, which directly affects the interpretation of T-cell-derived interferon signals in the tumor cell (52).

#### 1.9.3 Safety considerations and normal-tissue risk

A recurring feature of the targets discussed above is that none of them is a tumor-restricted protein. Splicing factors, m6A writers and erasers, ADAR1, NAT10 and the NMD machinery are constitutive components of RNA metabolism that operate in every nucleated cell, including the immune cells on which the therapeutic effect depends. Therapeutic index therefore cannot be inferred from target expression alone, and the safety questions differ in kind from those raised by antibody or cell therapies directed at surface antigens. **Table 6** summarizes the principal translational challenge associated with each approach; the paragraphs below consider the toxicities that follow specifically from modulating these pathways systemically. For ADAR1, human genetics already defines the risk. Loss-of-function mutations cause Aicardi–Goutieres syndrome, a severe autoinflammatory encephalopathy driven by uncontrolled type I interferon signaling, and Adar1 deletion is embryonically lethal in mice (166, 167). The antitumor rationale and the toxicity therefore arise from the same mechanism, which places a hard constraint on the achievable therapeutic window for systemic inhibition. Two considerations may widen it. First, ADAR1 acts both through A-to-I editing and through editing-independent RNA binding, so agents targeting the catalytic domain and agents degrading the protein should not be assumed to share a safety profile (165). Second, indirect strategies acting downstream, such as CBL0137, or destabilizing approaches such as ATRA, may separate antitumor activity from constitutive interferon suppression, although this remains to be shown clinically (83, 168).

For METTL3, the principal concern is that m6A is the most abundant internal mRNA modification and influences splicing, export, translation and decay across the transcriptome (148–150). Inhibition is therefore systemic by design rather than by accident, and effects on normal hematopoiesis and other rapidly renewing tissues require careful monitoring. A second and less widely discussed risk is directional. METTL3 is oncogenic in acute myeloid leukemia, glioblastoma, hepatocellular carcinoma and colorectal cancer, but tumor-suppressive in triple-negative breast cancer, where reduced expression promotes metastasis (152, 153, 183). An inhibitor that is efficacious in one setting could in principle be counterproductive in another, so patient selection is a safety requirement rather than only an efficacy optimization. The same context-dependence applies to erasers and readers, whose effects diverge across tumor types and between tumor and immune compartments (74–76, 163).

NMD inhibition raises a distinct problem. The therapeutic aim is to stabilize premature-termination-codon-containing transcripts so that cryptic neoantigens are translated and presented, but NMD does not act selectively on tumor transcripts. Inhibition stabilizes PTC-containing and NMD-sensitive transcripts genome-wide, including in normal tissue, and NMD has essential physiological roles in embryonic development, stem-cell differentiation, spermatogenesis, circadian regulation and the unfolded-protein and integrated stress responses (175–177). There is also a tumor-intrinsic hazard, since the same intervention that unmasks neoantigens may stabilize pro-tumorigenic transcripts (171, 178). The most credible mitigation is genotype-directed use rather than broad application: SF3B1- and U2AF1-mutant cells show selective dependency on NMD, which converts a general pathway inhibitor into a synthetic-lethal strategy in a defined population (180). NAT10 has the thinnest safety evidence of the targets considered here, and this should be stated plainly rather than assumed favorable. Preclinical data indicate that NAT10 inhibition increases dsRNA accumulation, type I interferon signaling and CD8+ T-cell activation (65), but ac4C is deposited on rRNA and tRNA as well as mRNA, so inhibition is not mRNA-selective and effects on ribosome biogenesis and translational fidelity in normal tissue have not been characterized. Delivery modality contributes an independent layer of risk. Cas13-based approaches can produce collateral, sequence-independent cleavage of bystander transcripts after target engagement, which depletes endogenous RNAs and activates stress and apoptotic responses; expression control such as negative autoregulation mitigates but does not eliminate this (123, 124). Antisense oligonucleotides raise different questions, principally biodistribution beyond liver and lymphoid tissue and the consequences of repeat dosing for an effect that is transient by design (125). These issues are considered in section 1.8.1 and are not restated here.

Taken together, these considerations argue that the feasibility of RNA-processing-targeted therapy will depend less on target validation than on achieving selectivity. Because the targets are housekeeping machinery, therapeutic index is most likely to come from tumor-selective delivery, from genotype-defined synthetic lethality, or from intermittent rather than continuous exposure, rather than from differential target expression between tumor and normal tissue. Trial designs in this class should therefore incorporate interferon and autoimmunity monitoring, hematologic safety endpoints, and biomarker-defined enrollment from the earliest phases.

## 2 Discussion

Target discovery in cancer immunotherapy has long been shaped by genomic alterations. Somatic mutations, gene fusions, tumor mutational burden, and predicted HLA-binding neoantigens have been central to checkpoint inhibitor response and personalized vaccine strategies. However, the studies discussed here show that tumor immune visibility is determined not only by the genome but also by RNA processing after transcription.

Alternative splicing, APA, RNA editing, epitranscriptomic modifications and NMD shape antigenic repertoire, checkpoint expression, innate sensing, and tumor-microenvironment interactions. Thus, mRNA processing should not be treated as an auxiliary molecular detail but as a central regulatory step for target discovery and treatment resistance. One of its strongest contributions is expansion of the mutation-based neoantigen paradigm. Tumor-specific splicing events, new junctions, retained introns, and tumor-specific isoforms can create targets even in cancers with low tumor mutational burden (7, 8, 24, 25, 107). Yet RNA-level candidates must still be validated at the protein or HLA-peptide level (17, 18).

mRNA processing also explains the post-transcriptional architecture of immune escape, because several resistance mechanisms operate after transcription and would be missed by DNA-level analysis alone. PD-L1 3′UTR disruption, DDX3–PD-L1 3′UTR interaction, the METTL3/IGF2BP1–PD-L1 m6A axis, and ADAR1-mediated dsRNA suppression show that similar immune-resistance phenotypes can be supported through different RNA layers (11, 39, 55, 68). These mechanisms can be therapeutically linked. ADAR1, NAT10 or NMD targeting may create viral mimicry-like states, but whether this becomes CD8+ T-cell infiltration or chemokine-mediated myeloid suppression depends on the tumor chemokine map (12, 21, 65, 86–89, 91). RNA processing-targeted therapies should therefore be considered with checkpoint blockade, chemokine receptor modulation, and mRNA vaccines. The most effective combinations will probably be those that increase immune visibility while also controlling which immune cells are recruited into the tumor.

Future work should prioritize multilayer validation: RNA-seq for candidates, long-read sequencing for isoform structure, immunopeptidomics for MHC presentation, single-cell/spatial omics for cell type and tissue context, and functional T-cell assays for immunogenicity (13–18, 22, 106, 126–131). Without this chain, clinical success will remain limited. At the same time, mRNA processing is becoming part of therapy design itself, not only a biological mechanism to be observed. mRNA vaccines, mRNA-encoded antibodies, mRNA-CAR T cell engineering and extracellular vesicle-mediated mRNA delivery turn RNA stability, UTR architecture, translational efficiency and innate-sensing balance into pharmaceutical parameters (83–87).

## 3 Conclusion

mRNA processing should no longer be viewed only as basic RNA biology in cancer immunotherapy. Post-transcriptional mechanisms directly affect how tumors are perceived by the immune system, which antigens are presented, how checkpoint signals are regulated, how innate RNA alarms are suppressed, and how resistance develops. Alternative splicing expands mutation-based neoantigen discovery through tumor-specific isoforms and public splicing neoantigens. APA, and 3′UTR remodeling can support immune escape by releasing checkpoint molecules such as PD-L1 from miRNA/RBP control. RNA editing, especially through ADAR1, limits innate immune sensing by silencing endogenous viral-like RNA signals. m6A, ac4C, and other epitranscriptomic modifications produce context-sensitive effects on RNA stability, translation, antigen presentation, and checkpoint response. NMD degrades abnormal transcripts, providing quality control but also limiting potential neoantigen visibility.

These mechanisms form the intermediate layer that converts tumor genomic information into immunological outcome. Future immunotherapy target discovery cannot rely only on DNA mutations or total gene expression, because neither fully captures isoform structure, RNA stability or antigen presentation. It will require integrated approaches combining exon/junction-level transcriptomics, long-read sequencing, RNA modification mapping, 3′UTR/APA analysis, RNA editing profiles, NMD activity scores, immunopeptidomics, single-cell/spatial omics and functional T-cell validation.

Therapeutically, mRNA processing offers two-sided opportunities: splicing-derived, NMD-derived, or editing-derived antigens can provide targets for vaccine-, TCR-T- or CAR-based therapies, while modulation of ADAR1, SMG1/SMG5, METTL3/METTL14, YTHDF1, NAT10 or 3′UTR-RBP interactions can make tumors more immunologically visible. Clinical success will depend on managing tumor versus normal specificity, cell-type context, HLA restriction, heterogeneity, antigen-presentation capacity, chemokine direction and toxicity. This is why RNA-derived candidates should be treated as hypotheses that require mechanistic and functional validation, rather than as ready therapeutic targets. In conclusion, mRNA processing is not a passive discovery area but an active biological control layer that can guide new immunotherapeutic strategies for tumors with low mutational burden or checkpoint inhibitor resistance.
